# A High Frequency of HIV-Specific Circulating Follicular Helper T Cells Is Associated with Preserved Memory B Cell Responses in HIV Controllers

**DOI:** 10.1128/mBio.00317-18

**Published:** 2018-05-08

**Authors:** M. Claireaux, M. Galperin, D. Benati, A. Nouël, M. Mukhopadhyay, J. Klingler, P. de Truchis, D. Zucman, S. Hendou, F. Boufassa, C. Moog, O. Lambotte, L. A. Chakrabarti

**Affiliations:** a Pasteur Institute, Viral Pathogenesis Unit, Paris, France; b INSERM U1108, Paris, France; c INSERM UMR_S1109, Center for Research in Immunology and Hematology, Medicine Faculty, Strasbourg Translational Medicine Federation (FMTS), Strasbourg University, Strasbourg, France; d AP-HP, Infectious and Tropical Diseases Department, Raymond Poincaré Hospital, Garches, France; e HIV Unit, Foch Hospital, Suresnes, France; f INSERM U1018, Center for Research in Epidemiology and Population Health (CESP), Le Kremlin-Bicêtre, France; g INSERM U1184, Center for Immunology of Viral Infections and Autoimmune Diseases, Le Kremlin-Bicêtre, France; h AP-HP, Department of Internal Medicine and Clinical Immunology, University Hospital Paris Sud, Le Kremlin-Bicêtre, France; i Université Paris Sud, UMR1184, Le Kremlin-Bicêtre, France; j CEA, DSV/iMETI, Division of Immuno-Virology, IDMIT, Fontenay-aux-Roses, France; Icahn School of Medicine at Mount Sinai; Icahn School of Medicine at Mount Sinai

**Keywords:** B lymphocytes, HIV controllers, MHC-II tetramers, T follicular helper, T lymphocytes, human immunodeficiency virus

## Abstract

Follicular helper T cells (Tfh) play an essential role in the affinity maturation of the antibody response by providing help to B cells. To determine whether this CD4^+^ T cell subset may contribute to the spontaneous control of HIV infection, we analyzed the phenotype and function of circulating Tfh (cTfh) in patients from the ANRS CO21 CODEX cohort who naturally controlled HIV-1 replication to undetectable levels and compared them to treated patients with similarly low viral loads. HIV-specific cTfh (Tet^+^), detected by Gag-major histocompatibility complex class II (MHC-II) tetramer labeling in the CD45RA^−^ CXCR5^+^ CD4^+^ T cell population, proved more frequent in the controller group (*P* = 0.002). The frequency of PD-1 expression in Tet^+^ cTfh was increased in both groups (median, >75%) compared to total cTfh (<30%), but the intensity of PD-1 expression per cell remained higher in the treated patient group (*P* = 0.02), pointing to the persistence of abnormal immune activation in treated patients. The function of cTfh, analyzed by the capacity to promote IgG secretion in cocultures with autologous memory B cells, did not show major differences between groups in terms of total IgG production but proved significantly more efficient in the controller group when measuring HIV-specific IgG production. The frequency of Tet^+^ cTfh correlated with HIV-specific IgG production (*R* = 0.71 for Gag-specific and *R* = 0.79 for Env-specific IgG, respectively). Taken together, our findings indicate that key cTfh-B cell interactions are preserved in controlled HIV infection, resulting in potent memory B cell responses that may play an underappreciated role in HIV control.

## IMPORTANCE

The rare patients who spontaneously control HIV replication in the absence of therapy provide a unique model to identify determinants of an effective anti-HIV immune response. HIV controllers show signs of particularly efficient antiviral T cell responses, while their humoral response was until recently considered to play only a minor role in viral control. However, emerging evidence suggests that HIV controllers maintain a significant but “silent” antiviral memory B cell population that can be reactivated upon antigenic stimulation. We report that cTfh help likely contributes to the persistence of controller memory B cell responses, as the frequency of HIV-specific cTfh correlated with the induction of HIV-specific antibodies in functional assays. These findings suggest that T follicular help may contribute to HIV control and highlight the need for inducing such help in HIV vaccine strategies that aim at eliciting persistent B cell responses.

## INTRODUCTION

Follicular helper T cells (Tfh) were identified in 2000 as the key helper CD4^+^ T cell population responsible for providing help to B cells (1, 2). Tfh are required for the maturation of high-affinity antibodies against T-cell-dependent antigens and for the development of germinal centers (GC), both in mice and humans (for reviews, see references 3 to 5). The Tfh form conjugates with B cells through cognate interactions dependent on T cell receptor (TCR)/peptide-major histocompatibility complex class II (MHC-II) antigen recognition and of costimulatory molecules. These interactions are needed to trigger immunoglobulin (Ig) class switching and are at the core of the selection process that promotes the survival of high-affinity Ig-producing B cells. Tfh can be identified by the expression of the CXCR5 chemokine receptor, which drives their localization to GC, and by a high expression of the regulatory marker PD-1, which may reflect ongoing activation through the TCR (1, 2). Tfh provide help to B cells through a series of costimulatory molecules (CD40L, ICOS, SLAM, CD28, OX40, and BTLA) and by secreting the cytokine interleukin-21 (IL-21) (4, 6). A defining transcription factor of the Tfh population is Bcl-6, which is required for high CXCR5 expression (7), though Bcl-6 needs to act in concert with a series of other transcription factors such as c-Maf, Ascl2, IRF4, BATF, and TCF-1 to confer full Tfh function (8–11). Tfh differentiation is finely regulated and not binary, as Tfh can show a degree of plasticity and share differentiation markers with other T helper types (12).

The circulating population of CXCR5^+^ CD4^+^ T cells, which represents about 10 to 15% of the blood CD4^+^ T cell pool in humans, was shown to share functional characteristics with Tfh that reside in lymphoid tissues. In particular, blood CXCR5^+^ CD4^+^ T cells have the capacity to provide help to B cells and promote IgG and IgA secretion in CD4/B cell coculture assays, thus demonstrating bona fide Tfh function (13, 14). These cells, termed circulating Tfh, or cTfh, are phenotypically distinct from the Tfh found in GC, with a low expression of costimulatory markers such as ICOS and PD-1 and of the transcription factor Bcl-6. The cTfh population is in a more resting state than GC Tfh, but there are indications that cTfh reacquire typical Tfh markers such as Bcl-6, ICOS, and PD-1 after *in vitro* activation (14) or *in vivo* reactivation following vaccination (15). Whether cTfh represent short-lived effector Tfh recently released from lymphoid organs or long-lived recirculating memory Tfh is still debated (16). However, there is a clear link between the circulating and lymphoid Tfh populations as both are increased in human and murine models of autoimmunity (17, 18). In addition, activated cTfh increase in frequency at peak response after influenza vaccination and correlate in frequency with the titers of the specific antibody response (15, 19). Recently, PD-1^+^ cTfh were shown to be clonally related to tonsillar GC Tfh through TCR clonotypic analysis, establishing the link between the two populations (20). In the same study, activated ICOS^+^ PD-1^+^ cTfh induced upon HIV (human immunodeficiency virus) vaccination were found to be clonally related to the resting PD-1^+^ cTfh population detected postvaccination, suggesting that cTfh persist as circulating memory cells in humans (20). In addition, a recent study in mice showed that GC Tfh could be released from lymphoid organs to the circulation upon blocking interactions with cognate antigens, revealing a dynamic exchange between the lymphoid and circulating Tfh pools (21). Of note, HIV-specific cTfh defined by IL-21 production were induced at higher levels by the partly efficacious RV144 HIV vaccine than by other candidate HIV vaccines that had shown no efficacy, suggesting a key role for the Tfh response in inducing protective antibodies directed at HIV (22). Recent studies have also reported an association between the proportion of PD-1^+^ cTfh or PD-1^+^ CXCR3^−^ cTfh and the induction of broadly neutralizing antibodies in HIV-infected patients, pointing to the relevance of cTfh studies for understanding the development of efficient antibody responses in the context of a chronic viral infection (23, 24).

Though Tfh help is thought to be essential to the maturation of antiviral humoral responses, Tfh also seem to contribute to HIV pathogenesis, through the promotion of abnormal B cell activation and HIV replication. The frequency of Tfh cells is markedly increased in lymph nodes of patients with progressive HIV infection (25, 26), in a context where other CD4^+^ T cell subsets show exhaustion and depletion. A possible reason may be that Tfh preferentially differentiate in situations of high antigenemia, when they receive strong and persisting signals through the TCR (27, 28). This notion is compatible with the observation that Tfh abundance generally increases in chronic, nonresolving viral and bacterial infections (4). Increases in IL-6 levels, which are a hallmark of abnormal immune activation, may also contribute to Tfh amplification (29). The predominance of Tfh helps explain the lymph node hyperplasia and the nonspecific hypergammaglobulinemia that are characteristic of progressive HIV and SIV (simian immunodeficiency virus) diseases. The frequency of HIV-specific cells among the Tfh population appears quite high, with median values of Gag-specific cells above 1% in viremic patients (25, 26), suggesting that HIV directly contributes to the amplification of Tfh responses. Importantly, Tfh also represent a major reservoir of HIV- or SIV-infected CD4^+^ T cells within lymphoid organs, with a higher viral DNA content than other CD4^+^ T cell subsets (26, 29–31). An intrinsically higher susceptibility of Tfh to HIV infection, possibly due to the repression of antiviral resistance genes by Bcl-6, could account for the high Tfh infection rate (26, 32). The scarcity of cytotoxic CD8^+^ T cells that traffic to GC may also limit the elimination of infected Tfh (33). Studies in the SIV model suggest that GC constitute viral sanctuaries even in macaques that naturally control viral replication in the periphery (34). Tfh also remain the most prominent HIV reservoir in lymph nodes of treated patients after viremia suppression, which poses a major obstacle to viral eradication (35, 36).

Several lines of evidence suggest that Tfh function is suboptimal in progressive HIV and SIV infections. Multiple studies have shown that the development of antibody responses to vaccines or other pathogens is impaired in HIV-infected patients (37, 38). For instance, the proportion of nonresponders to influenza vaccination is higher among HIV-infected individuals, and cTfh capacity to promote the secretion of influenza virus-specific antibodies by memory B cells appears impaired in the nonresponders (15). Mechanistically, negative regulation via the PD-1/PD-L1 axis has been proposed to play a role in Tfh dysfunction, as memory B cells from HIV-infected patients showed overexpression of the PD-L1 ligand, which limited functional responses in cognate PD-1hi Tfh (39). Increased PD-L1 expression by dendritic cell (DC)-like cells has also been reported in the lymph nodes of macaques with progressive SIV infection, suggesting that abnormal PD-L1 expression may also perturb the priming of Tfh by antigen-presenting cells (33). The facts that memory B cells show signs of impaired differentiation in progressive HIV infection and that HIV-specific memory B cells in particular appear depleted in patients with advanced infection are compatible with a loss of Tfh help (38, 40). In addition, the antibody response directed at HIV shows clear signs of impairment. A key observation is that antibodies able to efficiently neutralize autologous HIV strains take about 2 to 3 years to emerge in most patients (41, 42). These antibodies do not appear to bring much clinical benefit to patients, as HIV repeatedly and continuously escapes the antibody response through Env mutations (43). Antibodies able to neutralize a large spectrum of HIV variants, the so-called broadly neutralizing antibodies (bNAbs), occur only rarely (42, 44). Importantly, sequencing of Ig variable regions showed that bNAbs are highly hypermutated and hence highly dependent on Tfh help (44, 45). This suggests that the consecutive cycles of B cell-Tfh interactions required for extensive somatic hypermutation of anti-HIV antibodies are impaired or interrupted in most patients with progressive HIV disease.

HIV controllers (HIC), also called elite controllers, are rare patients who spontaneously control HIV replication in the absence of antiretroviral therapy (46, 47). Fewer than 0.5% of HIV-1-seropositive individuals maintain a controller status, as defined by a viral load of <50 copies of HIV-1 RNA/ml over 5 years, but these individuals have a very low risk of progression to AIDS (48). HIV controllers provide a unique model to elucidate the determinants of an efficient immune response directed at HIV. Multiple studies have documented that HIV controllers have a particularly efficient cellular immunity directed against HIV, with highly cytotoxic CD8^+^ T cells able to suppress viral replication in CD4^+^ target cells (49, 50), and polyfunctional CD4^+^ T cells endowed with high proliferative capacity and high antigen sensitivity (47, 51–53). In contrast, the possible contribution of the humoral arm of the immune response to HIV control has not been emphasized, as controllers do not harbor high neutralizing antibody titers (46). Multiple studies have demonstrated that the emergence of bNAbs requires relatively high viremia, high viral diversity, and multiple rounds of viral escape to emerge (41). While bNAbs are extremely promising tools for HIV prevention and immunotherapy, they seem to provide only limited benefit to the patients who naturally develop them, due to continuous viral escape (41, 43). In the absence of autologous virus neutralization, high bNAb titers may thus reflect the persisting but failing efforts of the humoral response at containing HIV replication and diversification. There are indications that anti-HIV antibody levels parallel HIV viremia in patients with low viral loads (54), suggesting that low NAb titers in HIV controllers may actually reflect efficient viral containment. However, interest in the involvement of the humoral response in HIV control has been revived, with the recent realization that HIV-specific memory B cell responses are actually strong in HIV controllers and are thus disconnected from circulating NAb titers. Analyses of memory B cells based on HIV-specific Ig production after restimulation in limiting dilution cultures or in B cell enzyme-linked immunosorbent spot assays (ELISpot) (40, 55–57) or on direct labeling with Env-derived protein probes (56) all revealed potent responses in HIV controllers, which surpassed those observed in treated patients or in viremic patients with advanced HIV infection. Thus, an emerging notion is that the preserved memory B cell population in HIV controllers has the capacity to transiently reactivate and produce NAbs upon HIV replication episodes, which may contribute to the suppression of viremia.

Preserved Tfh function may help explain the persistence of a large HIV-specific memory B cell population in controlled HIV infection. To test this notion, we set out to characterize the HIV-specific cTfh population of HIV controllers using the MHC-II tetramer technology, which enabled a direct quantitation and phenotyping of this rare CD4^+^ T cell population without perturbations associated with *in vitro* culture. We compared HIV controllers with stringent viral control (<50 copies of HIV-1 RNA/ml) for over 5 years to patients who had received efficient antiretroviral therapy (ART) for over 5 years, ensuring that functional differences between the two groups would not result primarily from differences in viral antigen loads. This approach revealed that the HIV-specific cTfh population was more abundant in controllers and showed signs of chronic antigenic stimulation that remained lower than those seen in treated patients. Assays of Tfh function revealed an efficient induction of HIV-specific IgG in cTfh/memory B cell cocultures from controllers, while this response appeared defective for treated patients and could not be restored by cytokine supplementation. The frequency of HIV-specific cTfh correlated with IgG induction in the cocultures, supporting the notion of efficient cTfh-B cell interactions in controlled HIV infection.

## RESULTS

### The cTfh population displays a central memory phenotype with high PD-1 expression.

**FIG 1:**
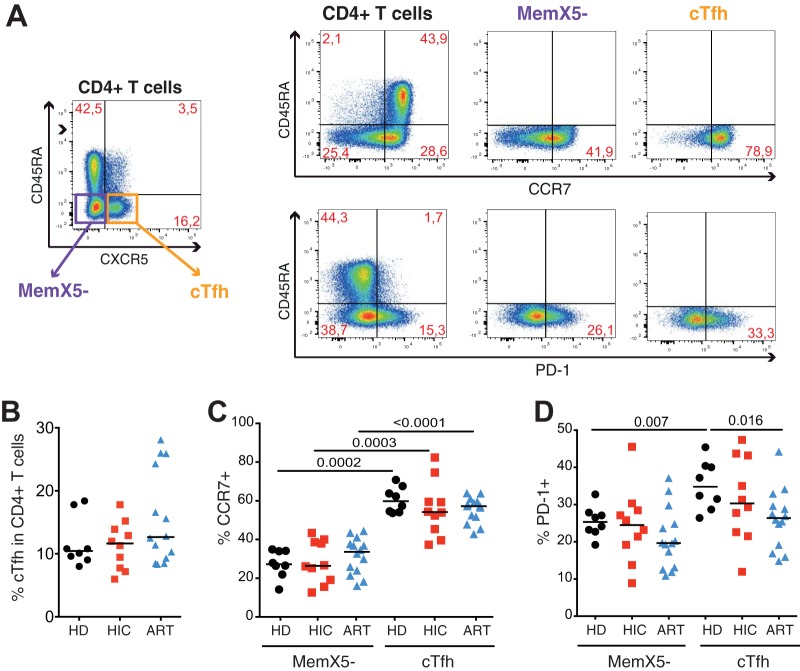
Phenotyping of the total cTfh population. Flow cytometry analysis of CD4^+^ T cells derived from healthy donors (HD, *n* = 8), HIV controllers (HIC, *n* = 10), and treated patients (ART, *n* = 14). (A) Representative plots depicting the gating of subsets analyzed in the CD4^+^ T cell population. (Left) CD4^+^ T cells were initially gated as viable CD3^+^ CD20^−^ CD14^−^ CD8^−^ CD4^+^ cells and were then analyzed for the following subsets: MemX5^−^ (CD45RA^−^ CXCR5^−^; purple square) and cTfh (CD45RA^−^ CXCR5^+^; orange square). The CD4^+^, MemX5^−^, and cTfh populations were then analyzed in relation to CD45RA and CCR7 expression (upper right panel) as well as CD45RA and PD-1 expression (lower right panel). (B) Comparison of the frequencies of cTfh in CD4^+^ T cells between healthy donor, HIV controller, and treated patient groups. (C and D) Comparison of the frequencies of CCR7^+^ (C) and PD-1^+^ (D) cells within the MemX5^−^ and cTfh subsets between healthy donor, HIV controller, and treated patient groups. Bars represent medians. Significant differences (*P* < 0.05) obtained with the Mann-Whitney U test are reported.

In a first analysis, we characterized the frequency and phenotype of the total cTfh population from healthy donors (HD, *n* = 8), HIV controllers (HIC group, *n* = 10), and treated patients (ART group, *n* = 14). Clinical characteristics of patient groups are reported in Table S1A in the supplemental material. The cTfh population was defined as the circulating CD3^+^ CD4^+^ CD45RA^−^ CXCR5^+^ T cell subset among live lymphocytes (Fig. 1A). The gating strategy used and examples of “fluorescence minus one” (FMO) control labeling are reported in Fig. S1. The phenotype of cTfh was compared to that of memory CD4^+^ T cells that did not express the CXCR5 homing marker (MemX5^−^). Flow cytometry analysis showed that the cTfh population represented close to 10% of the circulating CD4^+^ T cell population, irrespective of the HIV infection status (Fig. 1B). No significant differences in cTfh frequencies were observed between the 3 groups, though a greater individual heterogeneity was noted in the ART group, with a few treated patients showing cTfh frequencies of >25%. The cTfh population showed a predominantly central memory phenotype in the 3 groups, as indicated by a high percentage of CCR7 expression (medians, 44% to 54% [Fig. 1C]), which was significantly higher than that observed in the MemX5^−^ population (medians, 26% to 33%). The cTfh population from healthy donors expressed higher levels of the PD-1 marker than the MemX5^−^ population (medians, 35% versus 25%, *P* = 0.007) (Fig. 1D), compatible with the high PD-1 expression characteristic of tissue Tfh (58). Patient cTfh also showed a trend for higher PD-1 expression than in the MemX5^−^ population, which did not reach significance due to higher intragroup variability (Fig. 1D). Taken together, the cTfh population did not show signs of major perturbations in these patients with efficiently controlled viremia.

10.1128/mBio.00317-18.1 FIG S1  Gating strategy and FMO controls for cTfh phenotyping. (A) CD4^+^ T cells were analyzed by gating the singlet viable CD14^−^ CD19^−^ CD3^+^ CD4^+^ lymphocyte population. The gate for the CCR7 marker was set on a CD45RA/CCR7 dot plot, which enabled a clear distinction between naive (Nv; CD45RA^+^ CCR7^+^) and memory subsets. The three memory subsets including the central memory (CM; CDRA^−^ CCR7^+^), effector memory (EM; CD45RA^−^ CCR7^−^), and advanced effector (Eff; CD45RA^+^ CCR7^−^) subsets are reported. Similarly, gates for the PD-1 and CXCR5 markers were set on dot plots in relation to the CD45RA parameter, which enabled a clear distinction between the positive and negative populations (2 rightmost dot plots). (B and C) “Fluorescence minus one” (FMO) controls were obtained by omitting a single antibody from the labeling antibody cocktail. Analysis of FMO controls for CCR7 (B) and PD-1 (C) showed that omitting one marker did not significantly change the frequency of positive cells for other markers, confirming the validity of the compensation matrix and of the gating strategy. Download FIG S1, PDF file, 1.4 MB. Copyright © 2018 Claireaux et al. 2018 Claireaux et al. This content is distributed under the terms of the Creative Commons Attribution 4.0 International license.

10.1128/mBio.00317-18.9 TABLE S1  Clinical characteristics and HLA-DR typing of patients included in the study. Fifteen HIV controllers (HIC) and 15 treated patients (ART) were included in the study. (A) Summary of clinical characteristics of studied patients. Median values and ranges are reported. *, *P* values estimated with the Mann-Whitney U test are reported. N.S., not significant. N/A, not available. (B and C) Clinical characteristics and HLA-DR typing of HIC (B) and ART (C) patients. Patients included in the study were genotyped for HLA-DRB1. A subgroup of 10 HIC and 8 ART patients were analyzed with HLA-DR-matched MHC-II tetramers loaded with the Gag293 peptide. The tetramers used for this analysis are reported in the rightmost column. The following tetramers were used: HLA DRB1*0101 (DR1), DRB1*0401 (DR4), DRB1*0405 (DR4*), DBRB1*0701 (DR7), DRB1*1101 (DR11), DRB1*1302 (DR13), DRB1*1502 (DR15), and DRB5*0101 (DRB5). Download TABLE S1, PDF file, 0.04 MB. Copyright © 2018 Claireaux et al. 2018 Claireaux et al. This content is distributed under the terms of the Creative Commons Attribution 4.0 International license.

### Increased population of HIV-specific cTfh in HIV controllers.

**FIG 2:**
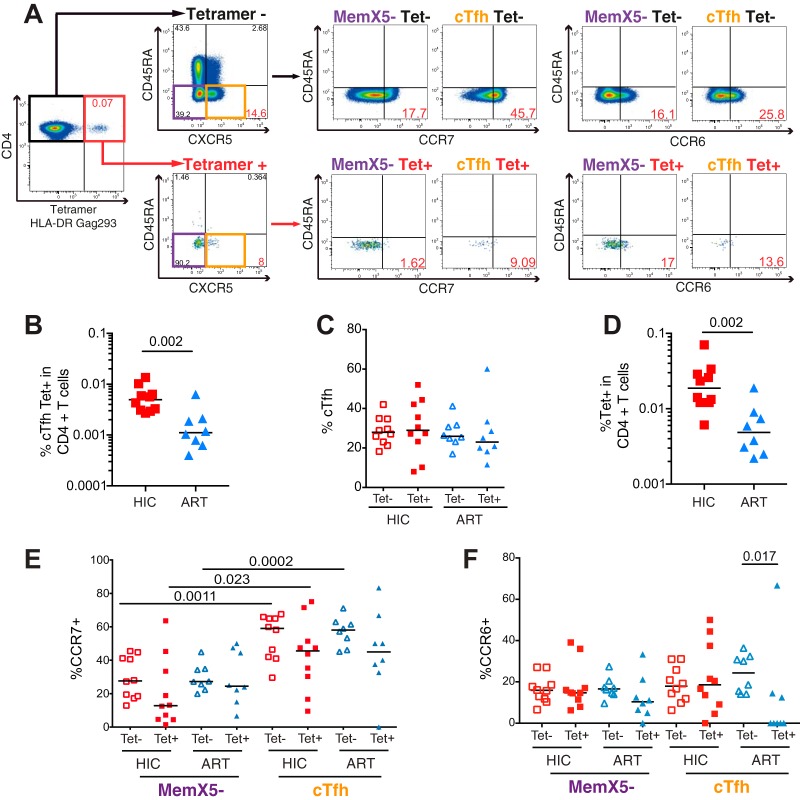
Phenotyping of HIV-specific cTfh cells. Phenotyping of CD4^+^ T cells derived from HIV controllers (HIC, *n* = 10) and treated patients (ART, *n* = 8) with Gag293-loaded MHC-II tetramers. (A) Gating strategy used to analyze HIV-specific CD4^+^ T cells, shown for one HIV controller. (Farthest left) Dot plot depicting the detection of Gag293-specific CD4^+^ T cells by MHC-II tetramer labeling, among total CD4^+^ T cells. (Middle left) Gates used to select Mem X5^−^ (CD45RA^−^ CXCR5^−^; purple) and cTfh (CD45RA^−^ CXCR5^+^; orange) in Gag293-specific (bottom) or nonspecific (top) CD4^+^ T cell populations. (Middle and right) MemX5^−^ and cTfh populations were then analyzed in relation to CD45RA versus CCR7 (middle panels) or CD45RA versus CCR6 (right panels). (B) Frequency of Gag293-specific cTfh cells in the HIC and ART groups. (C) Comparison of the frequency of cTfh cells in the memory CD45RA^−^ CD4^+^ T cell populations that are Gag293 specific (Tet^+^) and nonspecific (Tet^−^) in the HIC and ART groups. (D) Frequency of total Gag293-specific cells in CD4^+^ T cells of controllers and treated patients. (E) Frequency of CCR7^+^ cells in the cTfh and Mem X5^−^ cell populations: comparison between Gag293-specific (Tet^+^) and nonspecific (Tet^−^) CD4^+^ T cells in the HIC and ART groups. (F) Frequency of CCR6^+^ cells in the cTfh and Mem X5^−^ cell populations, analyzed as in panel E. Bars represent medians. Significant differences (*P* < 0.05) obtained with the Mann-Whitney U test are reported.

To characterize HIV-specific cTfh unperturbed by *in vitro* culture, we labeled them with MHC-II tetramers. The study focused on CD4^+^ T cells specific for the most immunoprevalent epitope in the HIV-1 proteome, Gag293, located at positions 293 to 312 in the HIV-1 capsid. Responses to this CD4 epitope are exceptionally prevalent, as they can be detected in close to 50% of treated patients and in >70% of HIV controllers (59–61). The Gag293 epitope shows broad HLA-DR cross-restriction (53), which enables the study of patients with varied HLA genotypes. Patients included in the tetramer study expressed at least one of the 7 following HLA-DR alleles: DRB1*0101, DRB1*0401, DRB1*0405, DRB1*0701, DRB1*1101, DRB1*1302, or DRB1*1502 (Table S1B and C). Labeling with the corresponding HLA-DR tetramers was performed on a minimum of 10^7^ peripheral blood mononuclear cells (PBMC) per sample, to take into account the preferential depletion of HIV-specific CD4^+^ T cells in the course of progressive HIV infection (62). The gating strategy used to analyze Gag293-specific cells is represented in Fig. 2A and S2.

10.1128/mBio.00317-18.2 FIG S2  Gating strategy for the analysis of HIV-specific CD4^+^ T cell populations. (A) Example of gating used to analyze HIV-specific CD4^+^ T populations in one HIV controller. (Left) HIV-specific CD4^+^ T cells were detected by labeling with an MHC-II tetramer loaded with the Gag293 peptide. (Right) Plots depicting how the gates were set for the analysis of CCR6, PD-1, and CCR7 expression in the Gag293-specific (bottom row) and nonspecific (top row) CD4^+^ T cell populations. The CXCR5, CCR6, PD-1, and CCR7 gates were set based on differential expression in the CD45RA^+^ and CD45RA^−^ populations. (B) Example of CCR6 expression analysis in Gag293-specific and nonspecific CD4^+^ T cells from a treated patient. (Left) Gating strategy used to analyze HIV-specific CD4^+^ T cells. (Top) Plot illustrating the detection of Gag293-specific CD4^+^ T cells by MHC-II tetramer labeling in a treated patient. (Bottom) Gates used to select the Mem X5^−^ (CD45RA^−^ CXCR5^−^; purple) and cTfh (CD45RA^−^ CXCR5^+^; orange) subsets in Gag293-specific (Tetramer +) and nonspecific (Tetramer −) CD4^+^ T cell populations. (Right) Plots comparing CCR6 expression in the MemX5^−^ (right) and cTfh (left) subsets, in both the nonspecific (Tet^−^, top) and specific (Tet^+^, bottom) CD4^+^ T cell populations. Download FIG S2, PDF file, 0.8 MB. Copyright © 2018 Claireaux et al. 2018 Claireaux et al. This content is distributed under the terms of the Creative Commons Attribution 4.0 International license.

Tetramer^+^ (Tet^+^) cells were readily detected in the cTfh population and were highly enriched in the memory compared to the naive CD4^+^ T cell population, as expected (Fig. 2A). The frequency of Tet^+^ cTfh in CD4^+^ T cells was 4.4 times higher in the HIC than in the ART group (medians, 0.0049% for HIC versus 0.0011% for ART; *P* = 0.002) (Fig. 2B), pointing to the preservation of Gag293-specific cTfh in HIV controllers. Of note, Gag293-specific Tet^+^ cells could not be detected in the blood of patients with persistent HIV replication (not shown). Analysis of the absolute count of specific cTfh in the circulation supported the notion of a preserved antiviral response in HIV controllers, with a median of 42 versus 4.9 cTfh/ml blood in the HIC and ART groups, respectively (*P* = 0.0005; data not shown). To determine whether this difference was due to an enrichment of Gag293-specific cells in the cTfh subset, we compared the proportions of cTfh in the Tet^+^ and Tet^−^ CD45RA^−^ CD4^+^ T cell populations (Fig. 2C). This analysis did not reveal significant differences, with a proportion of cTfh that ranged in median between 23% and 29% of memory CD45RA^−^ CD4^+^ T cells, in the Tet^+^ and Tet^−^ populations of the two groups. Rather, the frequency of Tet^+^ cells in the total CD4^+^ T cell population distinguished the HIC and ART groups, with a 3.8-fold increase of specific cells in HIV controllers (medians, 0.019% for HIC versus 0.0049% for ART; *P* = 0.002) (Fig. 2D). Thus, the increased frequency of HIV-specific cTfh in controllers reflected an overall higher frequency of HIV-specific cells, rather than a preferential enrichment of specific cells in the cTfh subset.

### Signs of antigenic activation in the HIV-specific cTfh population.

The CCR7 marker remained highly expressed in HIV-specific cTfh, indicating a predominant central memory phenotype (Fig. 2A and E). However, a trend for decreased CCR7 expression was observed in the specific cTfh populations, reflecting a partial shift to an effector memory phenotype. CCR7 expression remained significantly higher in the Tet^+^ cTfh than in the Tet^+^ MemX5^−^ population in the HIC group (*P* = 0.023), but this difference was lost in the ART group, with a high intragroup variability in both groups. The trend for CCR7 decrease raised the possibility that HIV-specific cTfh underwent a degree of chronic antigenic stimulation. This notion was further supported by analyses of the naive (Nv), central memory (CM), effector memory (EM), and effector (Eff) subsets defined by the combination of the CCR7 and CD45RA markers (gating shown in Fig. S1 and S2). Namely, HIV-specific CXCR5^+^ (X5^+^) cells showed a shift in subset hierarchy, with a significant increase in the EM subset (*P* < 0.05) compared to nonspecific X5^+^ cells, where CM cells predominated (Fig. S3). However, subset distribution did not show significant differences between patient groups. Of note, the Eff subset (CD45RA^+^ CCR7^−^) proved significantly decreased (*P* < 0.01) in the specific compared to the nonspecific X5^+^ population, pointing to a lack of advanced effector differentiation in HIV-specific cells.

10.1128/mBio.00317-18.3 FIG S3  Distribution of naive and memory subsets in Gag293-specific and nonspecific CD4^+^ T cells differs depending on CXCR5 expression. Gag293-specific (Tet^+^) and nonspecific (Tet^−^) CD4^+^ T cells expressing CXCR5 (X5^+^) or not (X5^−^) were analyzed for the distribution of the 4 following subsets: naive (Nv; CD4RA^+^ CCR7^+^), central memory (CM; CD45RA^−^ CCR7^+^), effector memory (EM; CD45RA^−^ CCR7^−^), and effector (Eff; CD45RA^+^ CCR7^−^). Analyses were carried out in cells from HIV controllers (HIC, *n* = 10) and treated patients (ART, *n* = 8). As no significant differences were found in subset distribution between the HIC and ART groups, data from the two groups were pooled and plotted together. (A) Nonspecific CXCR5^−^ CD4^+^ T cells. (B) Gag293-specific CXCR5^−^ CD4^+^ T cells. (C) Nonspecific CXCR5^+^ CD4^+^ T cells. (D) Gag293-specific CXCR5^+^ CD4^+^ T cells. *P* values for significant differences (*P* < 0.05) obtained by the Mann-Whitney U test between subsets on the same graph are reported on each graph. Significant intergraph differences obtained by the Mann-Whitney U test between Tet^−^ and Tet^+^ matching subsets are indicated by stars next to the subset name on panel C (Tet^−^ X5^−^ versus Tet^+^ X5^−^) or D (Tet^−^ X5^+^ versus Tet^+^ X5^+^): *, *P* < 0.05; **, *P* < 0.01; ***, *P* < 0.001. Download FIG S3, PDF file, 0.4 MB. Copyright © 2018 Claireaux et al. 2018 Claireaux et al. This content is distributed under the terms of the Creative Commons Attribution 4.0 International license.

The CCR6 marker was also analyzed, as expression of this chemoreceptor has been associated with efficient cTfh function (13). The CXCR3 marker, which is often used to define less efficient Th1-like cTfh, could not be analyzed in this study, as it was partially internalized under the tetramer labeling conditions. CCR6 was found to be expressed at comparable levels in the MemX5^−^ subsets of HIC and ART patients, in both the Gag293-specific and nonspecific cell populations (Fig. 2F). In contrast, CCR6 was found to be significantly decreased in the specific compared to the nonspecific cTfh subset in the ART group (*P* = 0.017). Specific cTfh from the ART group also showed a trend for lower CCR6 expression than did those of the HIC group (*P* = 0.066), pointing to a possible impairment of HIV-specific cTfh function in treated patients.

**FIG 3:**
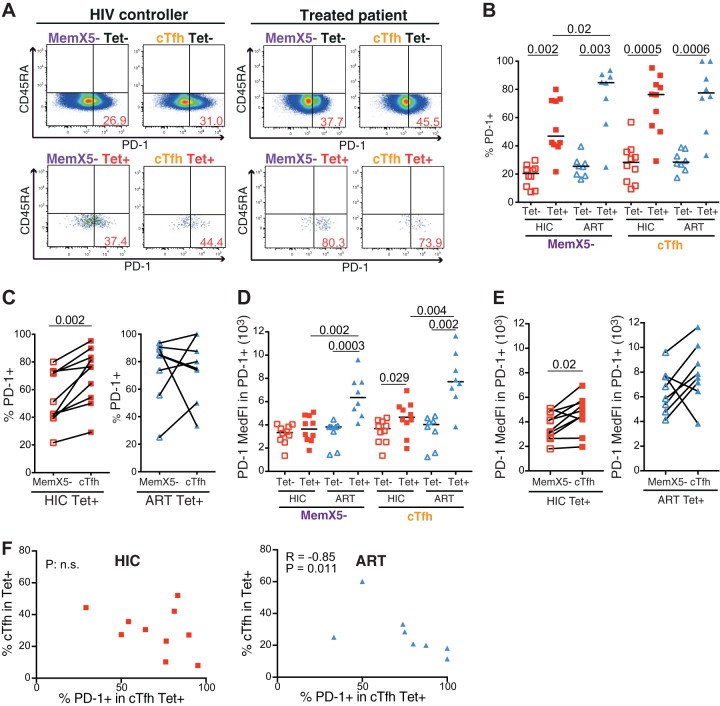
Analysis of PD-1 expression in HIV-specific cTfh cells. Phenotyping of Gag293-specific and nonspecific CD4^+^ T cells derived from HIV controllers (HIC, *n* = 10) and treated patients (ART, *n* = 8). The gating strategy used is identical to one described in the Fig. 2A legend. (A) Representative plots depicting the gating used for PD-1 analysis in CD4^+^ T cell populations. MemX5^−^ (left) and cTfh (right) populations were analyzed in relation to CD45RA versus PD-1 in Gag293-specific (bottom) and nonspecific (top) CD4^+^ T cell populations. Examples are shown for one HIV controller and one treated patient. (B) Frequency of PD-1^+^ cells in the cTfh and Mem X5^−^ cell populations: comparison between the Gag293-specific (Tet^+^) and nonspecific (Tet^−^) CD4^+^ T cell populations in the HIC and ART groups. (C) Comparison of the frequency of PD-1^+^ cells in paired cTfh and MemX5^−^ Gag293-specific populations from the HIC and ART groups. (D) Median florescence intensity (MedFI) of PD-1 expression in the PD-1^+^ subset of cTfh and Mem X5^−^ cell populations: comparison between Gag293-specific (Tet^+^) and nonspecific (Tet^−^) CD4^+^ T cell populations in the HIC and ART groups. (E) Comparison of the MedFI of PD-1^+^ cells in paired cTfh and MemX5^−^ Gag293-specific populations from the HIC and ART groups. (F) Correlation between the frequency of PD-1^+^ cells in the Gag293-specific cTfh population and the frequency of cTfh within the Gag293-specific population in the HIC (left) and ART (right) groups. Bars represent medians. Significant differences (*P* < 0.05) obtained by the Mann-Whitney U test (B and D), the Wilcoxon matched-pair statistical test (C and E), and Spearman’s rank correlation coefficient test (F) are reported.

A marked increase in PD-1 expression was noted in HIV-specific CD4^+^ T cells from controllers and treated patients, this increase being highly significant (*P* < 0.005) in both the cTfh and the MemX5^−^ subsets (Fig. 3A and B). As PD-1 induction is thought to result from TCR signaling upon cognate antigen recognition, these findings suggested that specific CD4^+^ T cells were chronically stimulated by Gag antigens, in spite of the very low viremia characteristic of both the controller and the treated patient groups. Further analyses of HLA-DR expression, as measured by quantitative real-time PCR (qPCR), confirmed that all Gag293-specific cells, in both the cTfh and MemX5^−^ subsets, were significantly more activated than their nonspecific counterparts, supporting the notion of chronic antigenic stimulation (Fig. S4A). An additional activation marker, FAS (FAS cell surface death receptor), also showed a trend for higher expression in Gag293-specific cells, except, interestingly, in the specific cTfh population of HIV controllers (Fig. S4B).

10.1128/mBio.00317-18.4 FIG S4  Expression of activation and exhaustion markers in HIV-specific and nonspecific CD4^+^ T cell subsets. HLA-DR (A), FAS (B), CTLA-4 (C), and LAG-3 (D) mRNA expression was measured in sorted Gag293-specific (Tet^+^) and nonspecific (Tet^−^) CD4^+^ T cell subsets. Reverse-transcribed mRNA was quantitated at the single-cell level by quantitative real-time PCR on a microfluidics C1 chip (Fluidigm), per manufacturer’s instructions. Gene expression normalized to that of the housekeeping gene glyceraldehyde-3-phosphate dehydrogenase (GAPDH) and multiplied by a factor of 10,000 is reported. The analysis was carried out on cells collected from 9 HIC and 9 ART patients. The number of cells analyzed was ≥140 for each group in the MemX5^−^ subset and ≥21 for each group in the cTfh subset. Violin plots visualize the distribution of the data set. Median values are indicated by red bars. The percentage of positive cells (with a normalized gene expression > 10) is indicated above each plot. *P* values obtained by the Mann-Whitney U test are reported. Download FIG S4, PDF file, 1.4 MB. Copyright © 2018 Claireaux et al. 2018 Claireaux et al. This content is distributed under the terms of the Creative Commons Attribution 4.0 International license.

HIV-specific cTfh expressed PD-1 at comparably high frequencies in the two groups (medians, 76.2% [HIC] versus 77.5% [ART] PD-1^+^ in Tet^+^ cTfh) (Fig. 3B), though individual variability in PD-1 induction remained high. In contrast, PD-1 induction was significantly higher in the specific MemX5^−^ subset of treated patients than in that of controllers (85.0% [ART] versus 42.2% [HIC] PD-1^+^ in Tet^+^ MemX5^−^; *P* = 0.02), reflecting a higher level of chronic immune activation and possibly exhaustion in the specific CD4^+^ T cell population of treated patients. These findings were confirmed in analyses that distinguished the memory subsets within the specific X5^+^ and X5^−^ populations (Fig. S5), with equivalent frequencies of PD-1 expression in the X5^+^ subsets of the two groups but a significantly higher PD-1 expression in the CM and EM X5^−^ subsets in the ART group. Paired analysis of the specific cTfh and MemX5^−^ subsets in each patient showed that the frequency of PD-1 expression was in all cases higher in the specific cTfh subset for controllers (*P* = 0.002) (Fig. 3C). This was not the case for treated patients, as specific MemX5^−^ cells in this group could express PD-1 at very high levels (>80%). Paired analyses of PD-1 expression in memory subsets within the specific X5^+^ and X5^−^ populations gave similar results (Fig. S5C and D) (significant differences between X5^−^ and X5^+^ Nv, CM, and EM subsets in the HIC but not in the ART group), reinforcing the notion that abnormal immune activation persisted in treated patients even after long-term antiretroviral therapy. This notion was further supported by an analysis of CD8^+^ T cells, which showed a higher frequency of PD-1 expression in treated patients than in HIV controllers (median PD-1^+^, 18.65% in ART versus 13.45% in HIC; *P* = 0.03; data not shown).

10.1128/mBio.00317-18.5 FIG S5  PD-1 expression in naive and memory subsets of Gag293-specific and nonspecific CD4^+^ T cells differs depending on CXCR5 expression. The frequency of PD-1-expressing cells was analyzed in subsets of Gag293-specific (Tet^+^) and nonspecific (Tet^−^) CD4^+^ T cells expressing CXCR5 (X5^+^) or not (X5^−^). PD-1 expression was measured in the 4 following subsets: naive (Nv; CD4RA^+^ CCR7^+^), central memory (CM; CD45RA^−^ CCR7^+^), effector memory (EM; CD45RA^−^ CCR7^−^), and effector (Eff; CD45RA^+^ CCR7^−^). Analyses were carried out in cells from HIV controllers (HIC, *n* = 10) and treated patients (ART, *n* = 8). Tet^+^ Nv and Eff data points with too few cells for analysis are not represented. (A) Nonspecific CD4^+^ T cells from HIC patients. (B) Nonspecific CD4^+^ T cells from ART patients. (C) Gag293-specific CD4^+^ T cells from HIC patients. (D) Gag293-specific CD4^+^ T cells from ART patients. *P* values for significant differences (*P* < 0.05) obtained by the Wilcoxon matched-pairs test between X5^−^ and X5^+^ matched subsets are reported on each graph. Significant intergraph differences obtained by the Mann-Whitney U test between Tet^+^ HIC and Tet^+^ ART matching subsets are indicated by stars next to the subset name on panel D; *, *P* < 0.05. Download FIG S5, PDF file, 0.4 MB. Copyright © 2018 Claireaux et al. 2018 Claireaux et al. This content is distributed under the terms of the Creative Commons Attribution 4.0 International license.

To further evaluate the extent of PD-1 induction, we monitored the median fluorescent intensity (MedFI) of PD-1 labeling in the subpopulations of PD-1-positive cells (Fig. 3D). This analysis showed that the intensity of PD-1 expression per cell was higher in specific cells from treated patients than in those of controllers, both in the MemX5^−^ (*P* = 0.002) and in the cTfh (*P* = 0.004) subsets. Paired analysis confirmed that specific cTfh expressed PD-1 at higher levels than specific MemX5^−^ cells in controllers, while the difference was blurred in treated patients, for whom both specific cTfh and non-cTfh expressed PD-1 at very high levels (Fig. 3E). Thus, PD-1 expression was induced in all Gag293-specific CD4^+^ T cells in both controllers and treated patients, indicating that these cells were not quiescent but rather engaged in an active antiviral immune response. In HIV controllers, PD-1 induction was more marked in the specific cTfh subset, which may reflect the activation of T follicular helper function. In treated patients, PD-1 induction was more generalized and reached higher levels, pointing to the persistence of abnormal immune activation.

Of note, in the ART group, the percentage of PD-1 induction in the specific cTfh population correlated inversely with the frequency of cTfh in the Tet^+^ population (*R* = −0.85, *P* = 0.011) (Fig. 3F). This correlation was not observed in the HIC group, as some controllers could have a significant PD-1 induction without a decrease in the proportion of specific cTfh. The inverse correlation observed for treated patients could reflect either a loss or a relocalization of specific cTfh upon increasing immune activation, raising the possibility of cTfh immune exhaustion. To further address this issue, we measured the expression of two immune exhaustion markers, CTLA-4 and LAG-3, by single-cell qPCR. These analyses showed generally low expression of both markers in cTfh and did not reveal significant differences between the HIC and ART groups (Fig. S4C and D). A trend for higher CTLA-4 and LAG-3 expression in specific than in nonspecific CD4^+^ T cell populations was noted, though it did not reach significance. Taken together, the analysis of PD-1, HLA-DR, and FAS expression was consistent with signs of chronic activation in HIV-specific cTfh, which were more marked in treated patients than in HIV controllers. However, the generally low expression of the CTLA-4 and LAG-3 markers, and their comparable expression in the two patient groups, suggested that HIV-specific cTfh were not in a state of advanced immune exhaustion.

### Low antibody neutralization titers in HIV controllers.

To evaluate the humoral response in controlled HIV infection, we first measured the concentration of HIV-specific antibodies in patient plasma by enzyme-linked immunosorbent assay (ELISA), using values normalized to total plasma IgG content (Fig. S6). Env-specific antibodies recognizing HIV-1 gp41, gp140, and gp160 were detected at comparable median values in the two groups, though individual variability was marked. Gag-specific antibodies directed at the p24 capsid protein showed a trend for higher concentrations in the HIC group, which did not reach significance (*P* = 0.2). Thus, the concentration of HIV-specific antibodies did not clearly distinguish the two patient groups.

10.1128/mBio.00317-18.6 FIG S6  HIV-specific antibodies in patient plasma. Antibodies specific for HIV-1 gp41 S30, gp140 and gp160 MN-LAI, and p24 Gag were measured by ELISA in patient plasma. The ratio of HIV-specific antibody concentration to the total IgG concentration in plasma, multiplied by 100, is reported. Ratios for undetectable HIV-specific antibodies were assigned a threshold value of 0.01. Median values are indicated by black bars. Download FIG S6, PDF file, 0.1 MB. Copyright © 2018 Claireaux et al. 2018 Claireaux et al. This content is distributed under the terms of the Creative Commons Attribution 4.0 International license.

We then analyzed the titers of neutralizing antibodies (NAbs) directed at HIV-1 in patient plasma (Table S2A). To this goal, serial dilutions of patient plasma were tested for their capacity to neutralize two tier 1 viral strains (63), six transmitted/founder (T/F) tier 2 strains, and one tier 3 T/F strain, as well as a negative-control murine leukemia virus (MLV) strain in a TZM-bl-based assay. Of note, plasma samples from treated patients neutralized the MLV control virus due to the presence of antiretroviral drugs and were not evaluated further. HIV controller plasma could in most cases neutralize the tier 1 HIV-1 strains tested but failed to neutralize the tier 2 and tier 3 T/F strains. Only one controller patient (HIC06) showed moderate neutralization titers (<100) against three of the tier 2 T/F strains tested. These moderate NAb levels appeared to be maintained over time, as testing of an HIC06 plasma sample obtained 8 months previously showed a similar neutralization pattern (Table S2B). Taken together, these data showed that NAb titers were low in HIV controllers, consistent with the literature (38, 64).

10.1128/mBio.00317-18.10 TABLE S2  Neutralizing capacity of HIV controller plasma. Titers of HIV-neutralizing antibodies were determined in a TZM-bl-based assay. Patient plasma samples were tested for their capacity to neutralize two tier 1 strains, six tier 2 strains, one tier 3 strain, and one control MLV strain. The reciprocal of the inhibitory dilution 50 (ID_50_) is reported. Values of >10 are considered significant. (A) Neutralizing titers obtained at inclusion in the study. (B) Neutralizing titers obtained for a subset of the same patients in retrospective plasma samples. The month of plasma collection is reported in the second column. ND, not done. Download TABLE S2, PDF file, 0.1 MB. Copyright © 2018 Claireaux et al. 2018 Claireaux et al. This content is distributed under the terms of the Creative Commons Attribution 4.0 International license.

### The cTfh CD4^+^ T cell subset provides efficient help to memory B cells *in vitro.*

As the antibody data did not preclude the possibility of a memory B cell response that could be reactivated upon stimulation, we developed an assay suited to the analysis of memory B cell-cTfh interactions. The assay relied on the coculture between sorted CD4^+^ T cell subsets and autologous memory B lymphocytes (BL). Sorted populations were >98% pure (see Fig. S7 for gating strategy). Sorted CD4^+^ T cell subsets included naive cells (CD45RA^+^ CCR7^+^), MemX5^−^ (CD45RA^−^ CXCR5^−^), and cTfh (CD45RA^−^ CXCR5^+^). Each sorted subset was cocultivated with autologous memory BL (CD3^−^ CD20^+^ CD27^+^) in the presence of superantigens, which induce polyclonal CD4^+^ T cell activation. CD4 helper function was analyzed by the induction of plasmablast differentiation, as measured by the number of CD38hi B cells in the coculture and by the amount of total immunoglobulin G (IgG) measured in coculture supernatants.

10.1128/mBio.00317-18.7 FIG S7  Gating strategy used for sorting CD4^+^ T cell and B cell subsets. Flow cytometry plots showing the gating strategy used for sorting cTfh cells (CD3^+^ CD20^−^ CD4^+^ CD45RA^−^ CXCR5^+^; orange), MemX5^−^ (CD3 CD20^−^ CD4^+^ CD45RA^−^ CXCR5^−^; purple), naive CD4^+^ T cells (CD3^+^ CD20^−^ CD4^+^ CD45RA^+^ CCR7^+^; green), and memory B cells (CD3^−^ CD20^+^ CD27^+^; blue) among live singlet PBMC. A representative example from a healthy donor sample pre- and postsorting is shown. Postsort sample plots are outlined with the color corresponding to the different cellular subsets. Download FIG S7, PDF file, 0.5 MB. Copyright © 2018 Claireaux et al. 2018 Claireaux et al. This content is distributed under the terms of the Creative Commons Attribution 4.0 International license.

**FIG 4:**
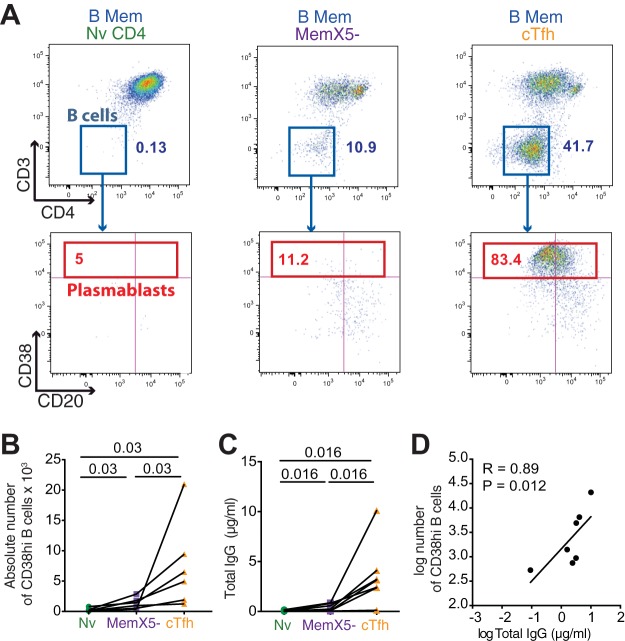
cTfh induction of B cell maturation and IgG production. Thawed PBMC from healthy donors were sorted into 3 CD4^+^ T cell subsets: cTfh (CD3^+^ CD20^−^ CD4^+^ CD45RA^−^ CXCR5^+^; orange), MemX5^−^ (CD3^+^ CD20^−^ CD4^+^ CD45RA^−^ CXCR5^−^; purple), and Nv or naive CD4^+^ T cells (CD3^+^ CD20^−^ CD4^+^ CD45RA^+^ CCR7^+^; green). The gating strategy is provided in Fig. S7. Each of these 3 subsets was then cocultured with autologous memory B cells (CD3^−^ CD20^+^ CD27^+^; blue) in the presence of superantigens. Cells and supernatants were harvested at days 7 and 12, respectively. (A) Representative flow cytometry plots depicting B cell maturation following 7 days of coculture. (Top panels) B cell gate (CD3^−^ CD4^−^); (bottom panels) analysis of plasmablasts (CD38hi) in the B cell population. (B) Absolute number of CD38hi B cells at day 7 of coculture with the 3 different CD4^+^ T cell subsets Nv (green), MemX5^−^ (purple), and cTfh (orange). (C) Total IgG secretion measured by ELISA in the supernatants of the cocultures at day 12. (D) Correlation between the number of CD38hi plasmablasts and total IgG production in the cocultures. Bars represent medians. Significant differences (*P* < 0.05) obtained by the Wilcoxon matched-pairs statistical test and Spearman’s rank correlation coefficient test are reported.

Functional analysis of healthy donor cells showed a clear hierarchy in the capacity of CD4^+^ T cell subsets to provide helper function to B cells, with cTfh proving more efficient than MemX5^−^ (*P* = 0.03), which were themselves more efficient than naive cells (*P* = 0.03) at inducing plasmablast differentiation (Fig. 4A and B). A similar hierarchy was observed in the production of IgG measured in day 12 supernatants: cTfh induced IgG secretion to a median concentration of 3 µg/ml and MemX5^−^ induced IgG to 0.5 µg/ml, while naive CD4^+^ T cells failed to induce IgG secretion from autologous BL (Fig. 4C). The differentiation of plasmablasts in the cocultures tightly correlated with the induction of IgG secretion (*R* = 0.89, *P* = 0.012) (Fig. 4D), indicating that both parameters could be used to monitor cTfh function. Taken together, the data obtained with healthy donor cells confirmed that cTfh could provide bona fide help to BL after *in vitro* stimulation, confirming that cTfh provide a relevant model to study Tfh function (13).

### Patient cTfh show a degree of functional impairment that can be reversed by IL-6 supplementation.

**FIG 5:**
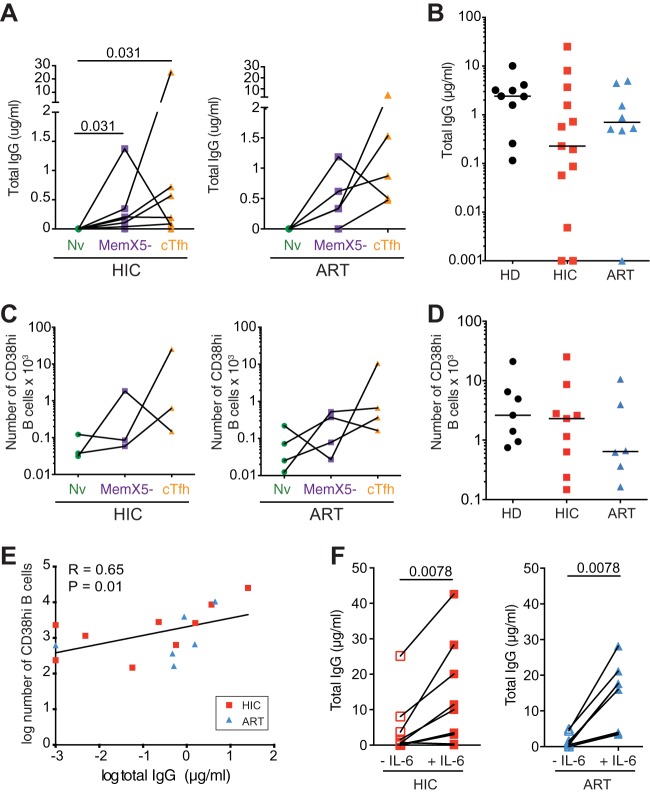
Analysis of patient cTfh-mediated help to autologous memory B cells. PBMC from healthy donors (HD), HIV controllers (HIC), and treated patients (ART) were sorted into 3 CD4^+^ T cell subsets and cocultured with memory B cells as described in the Fig. 4 legend. (A) Total IgG secretion was measured by ELISA in coculture supernatants at day 12. IgG secretion was compared in cocultures of memory B cells with naive CD4^+^ T cells (Nv), memory CXCR5^−^ CD4^+^ T cells (MemX5^−^), and cTfh in the HIC (*n* = 6, left) and ART (*n* = 5, right) groups. (B) Comparison of total IgG secretion in memory B cells/cTfh cocultures for the HD (*n* = 9), HIC (*n* = 13), and ART (*n* = 8) groups. (C) The B cell phenotype was analyzed at day 7 of coculture. The absolute numbers of plasmablasts (CD3^−^ CD4^−^ CD38hi) present in the different coculture systems (Nv, MemX5^−^, and cTfh) were compared in HIC (*n* = 3, left) and ART (*n* = 4, right) patients. (D) Comparison of the absolute number of plasmablasts in memory B cells/cTfh cocultures in the HD (*n* = 7), HIC (*n* = 9), and ART (*n* = 6) groups. (E) Correlation between the amount of secreted IgG and the absolute number of plasmablasts in memory B cells/cTfh cocultures from HIC (red squares) and ART (blue triangles) patients. (F) Effects of IL-6 addition on total IgG secretion in memory B cell/cTfh cocultures from HIC (left panel) and ART (right panel) patients. Bars represent medians. Significant differences (*P* < 0.05) obtained by the Wilcoxon matched-pair statistical test (A, C, and F), the Mann-Whitney U test (B and D), and Spearman’s rank correlation coefficient test (E) are reported.

The cTfh function showed signs of being perturbed in both the HIC and ART groups, as the hierarchy in helping capacity of the different CD4^+^ T cell subsets was not always conserved, with cTfh proving less efficient than MemX5^−^ cells at inducing IgG secretion for a few patients (Fig. 5A). As a consequence, IgG induction by the cTfh and MemX5^−^ subsets did not differ significantly, in the HIC as well as in the ART group. In addition, total IgG secreted in cTfh/BL cocultures showed a trend for decrease in the HIC and ART groups compared to the HD group, though the difference did not reach significance (Fig. 5B). Analysis of CD38hi plasmablasts in the cocultures led to similar conclusions, with in particular a trend for less efficient plasmablast differentiation in the ART group (Fig. 5C and D). Plasmablast induction still correlated with IgG secretion in patient cocultures, though the correlation was less tight than in healthy donor cocultures (*R* = 0.65, *P* = 0.01) (Fig. 5E), supporting the notion of perturbed cTfh-BL interactions in both patient groups.

As production of the IL-6 cytokine by plasmablasts was recently shown to support Tfh function (65), we set out to test the effect of IL-6 addition to the cTfh/BL cocultures. Interestingly, IL-6 supplementation restored cTfh function in the coculture of patient cells, as indicated by a significant increase in IgG production in both the HIC and ART groups (*P* = 0.0078 in both cases) (Fig. 5F). IL-6 restored IgG production to levels higher than those seen in cocultures from healthy donors (medians: HIC plus IL-6, 3.6 µg/ml; ART plus IL-6, 16.8 µg/ml; HD, 2.41 µg/ml). These findings suggested that the perturbation in patient cTfh function was not intrinsic but rather resulted from limiting or dysregulated cytokine secretion.

### High-level specific IgG production capacity in HIV controllers.

To evaluate the extent to which cTfh could stimulate HIV-specific memory B cell responses, we measured the production of IgG specific for HIV-1 p24 Gag or gp140 Env in cTfh/BL coculture supernatants. Recombinant proteins derived from HIV-1 subtype B, p24 Gag IIIB and the trimeric Env gp140 MN-LAI (66), were used as capture antigens as this subtype was the most prevalent among studied patients.

**FIG 6:**
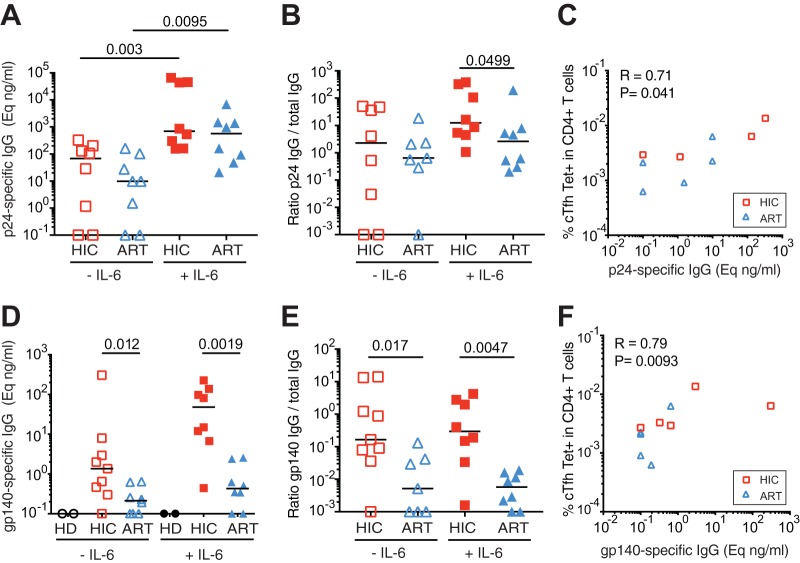
Analysis of HIV-specific IgG production stimulated by cTfh help. cTfh cells (CD3^+^ CD20^−^ CD4^+^ CD45RA^−^ CXCR5^+^) and memory B cells (CD3^−^ CD20^+^ CD27^+^) were sorted from patient PBMC samples and cocultured with superantigens in the presence or absence of IL-6. Supernatants were harvested at day 12. (A) Levels of Gag p24-specific IgG production in the presence or absence of IL-6, as measured by ELISA in cocultures from HIC (*n* = 8) and ART (*n* = 8) patients. (B) Ratio of p24-specific IgG to total IgG production in the presence or absence of IL-6 in HIC (*n* = 8) and ART (*n* = 8) patient cocultures. (C) Correlation between the level of p24-specific IgG production and the frequency of Gag293-specific cTfh in CD4^+^ T cells in HIC (red squares) and ART (blue triangles) patients. (D) Level of Env gp140-specific IgG production in the presence or absence of IL-6, as measured by ELISA in cocultures from HIC (*n* = 9) and ART (*n* = 8) patients. (E) Ratio of gp140-specific IgG to total IgG production in the presence or absence of IL-6 in HIC (*n* = 8) and ART (*n* = 8) patient cocultures. (F) Correlation between the level of gp140-specific IgG production and the frequency of Gag293-specific cTfh in CD4^+^ T cells in HIC (red squares) and ART (blue triangles) patients. Significant differences (*P* < 0.05) obtained by the Mann-Whitney U test (A, B, D, and E) and Spearman’s rank correlation coefficient test (C and F) are reported.

The cTfh/BL cocultures of controller patients showed a trend for higher production of anti-Gag IgG compared to those of treated patients, with a median 7-fold difference between the two groups (Fig. 6A, left). Supplementation with IL-6 significantly increased Gag-specific IgG production in both groups but dampened differences between the HIC and the ART groups (Fig. 6A, right). To take into account variations in total IgG production, we then computed the ratio of Gag-specific to total IgG (Fig. 6B). In this normalized analysis, Gag-specific IgG production was 3.5-fold higher in the HIC group in the absence of IL-6 and 4.8-fold higher in the HIC group in the presence of IL-6, with the difference reaching significance in the latter case (*P* = 0.0499). Interestingly, the frequency of Gag293-specific cTfh in the circulation correlated with Gag-specific IgG production in the cocultures (*R* = 0.71, *P* = 0.041) (Fig. 6C), supporting the notion that Tfh function contributed to the persistence of HIV-specific memory B cell responses.

Analysis of Env gp140-specific IgG production in the cTfh/BL cocultures revealed more pronounced differences between the HIC and the ART groups. Env-specific IgG was produced in median at 6.4-fold-higher levels in the HIC than in the ART group (*P* = 0.012) (Fig. 6D). IL-6 supplementation did not abrogate but rather amplified the difference, with 113-fold-higher levels of Env-specific IgG in the HIC group (*P* = 0.0019). After normalizing for total IgG production, the differences in Env-specific IgG production between the HIC and the ART groups were 32-fold in the absence of IL-6 (*P* = 0.017) (Fig. 6E) and were increased to 51-fold in the presence of IL-6 (*P* = 0.0047). It was striking that IL-6 supplementation had little effect on the synthesis of Env-specific IgG in the ART group, while total and Gag-specific IgG production responded to IL-6 supplementation for the same patients. This observation was confirmed by an analysis of the ratios of IgG produced in the presence and absence of IL-6, which were significantly lower for Env-specific IgG than for total or Gag-specific IgG in the ART group (Fig. S8). Of note, the frequency of Gag293-specific cTfh in the circulation showed a strong correlation with Env-specific IgG production in the cocultures (*R* = 0.79, *P* = 0.0093) (Fig. 6F), raising the possibility of intrastructural help provided by Gag-specific Tfh to Env-specific memory B cells (see Discussion). Taken together, these findings showed that treated patients, who had low frequencies of HIV-specific cTfh in the circulation, maintained defective memory B cell responses in spite of long-term therapy, with the defect being more marked for Env-specific than Gag-specific memory B cells. In contrast, key cTfh-B cell interactions were preserved in controlled HIV infection, resulting in potent memory B cell responses to both Gag and Env antigens.

10.1128/mBio.00317-18.8 FIG S8  Effect of IL-6 on IgG secretion in cTfh/B cell cocultures. cTfh and memory B cells were cocultivated in the presence of superantigens SEA and SEE for 12 days. Supernatants were assessed for the presence of total IgG, IgG specific for HIV Env gp140, and IgG specific for HIV capsid protein Gag p24. The ratio of IgG obtained with IL-6 supplementation to IgG obtained without IL-6 is reported for cocultures of cells from HIV controllers (HIC) and treated patients (ART). Significant differences (*P* < 0.05) obtained withe the Mann-Whitney U test are reported. Download FIG S8, PDF file, 0.1 MB. Copyright © 2018 Claireaux et al. 2018 Claireaux et al. This content is distributed under the terms of the Creative Commons Attribution 4.0 International license.

## DISCUSSION

This study provides evidence for the persistence of a highly functional HIV-specific cTfh population in controlled HIV infection. The use of MHC-II tetramer labeling enabled, for the first time to our knowledge, a direct quantitation and phenotypic characterization of HIV-specific cTfh in the circulation. This approach revealed significantly higher frequency and numbers of Gag-specific cTfh in HIV controllers than in treated patients, even though both groups were characterized by long-term viral suppression and very low antigenic loads. These findings are in line with the notion of a potent CD4-mediated adaptive response in controlled HIV infection and highlight in contrast the incomplete reconstitution of antiviral immunity in patients undergoing long-term antiretroviral therapy (47, 67). The high frequency of specific cTfh in the controller group suggests that successful HIV containment does not rely uniquely on Th1 responses, which are typically induced in the setting of chronic viral infections, but also involves Tfh help provided to the humoral arm of the immune response. This notion is supported by the potent induction of HIV-specific IgG detected in cTfh/B cell cocultures from controllers, as well as by the high frequency of memory B cells recently reported in the setting of controlled HIV infection (40, 55–57).

Unexpectedly, HIV-specific cTfh from both groups of patients showed signs of ongoing activation, as indicated by a marked increase in PD-1 and HLA-DR expression, and a moderate decrease of CCR7 expression compared to the total cTfh population. PD-1 induction is thought to reflect previous TCR engagement (68, 69), suggesting that cTfh encounter their cognate antigen, in this case Gag, even in situations of controlled viremia. Epigenetic studies indicate that PD-1 can in certain cases acquire constitutive expression in T cells after prolonged exposure to HIV (70), and we thus cannot rule out the possibility that high PD-1 expression in specific cTfh reflects a previous phase of viral replication, especially in the case of patients who had experienced high viral loads prior to treatment. However, the fact that PD-1 induction is also seen in cTfh of controllers, who did not experience massive HIV replication except possibly in acute infection, and the observation of a trend for CCR7 downregulation support the possibility of ongoing antigenic stimulation and partial shift toward an effector memory phenotype. This notion is further supported by the marked induction of HLA-DR expression, which points to a persistent activation of the HIV-specific cTfh population. Of note, expression of the CTLA-4 and LAG-3 markers remained low in HIV-specific cTfh, indicating that these cells were activated but not in an advanced stage of immune exhaustion. Given that tissue Tfh have been recently identified as the major HIV/SIV reservoir in lymphoid organs, in treated patients (25, 26, 35) but also in a simian model of controlled SIV infection (34), a likely possibility is that cTfh encountered their cognate antigen while recirculating through these organs. Systemically, HIV controllers show moderate but detectable signs of immune activation that subside upon initiation of antiretroviral therapy (71), suggesting that residual viral replication in lymphoid tissues drives immune activation, while the virus remains efficiently contained in the periphery. Another reason for the activation of specific cTfh in controllers could lie in their expression of particularly sensitive TCRs, as we recently reported that controller CD4^+^ T cells expressed TCRs of significantly higher affinity for Gag293–MHC-II complexes than those found in treated patients (53, 61). Thus, even minimal levels of viral antigens may trigger potent TCR signals in controller cTfh, which would promote their B cell helper function.

Of note, controller specific CD4^+^ T cells showed stronger PD-1 expression in the cTfh compartment than in the MemX5^−^ memory compartment, suggesting that cTfh may be more frequently exposed to viral antigen than specific cells that do not traffic through GC. In contrast, treated patients showed high PD-1 expression (≈75% PD-1^+^) in both cTfh and MemX5^−^ specific cells, which may point to the persistence of viral antigens in both GC and extrafollicular sites. It was also noteworthy that the intensity of PD-1 expression was significantly higher in the specific CD4^+^ T cell subsets of treated patients than of controllers, with the highest level of PD-1 expression per cell seen in the specific cTfh subset of treated patients. Given the negative regulatory function of the PD-1 receptor (68), these very high expression levels may impair cTfh function in treated patients. This notion is compatible with the report of a functional defect of Tfh function in HIV-infected individuals, due to overexpression of the PDL-1 ligand in lymph node B cells (39). In this study, the PD-1/PD-L1 interaction was shown to limit Tfh proliferation and helper function, which may help explain the persistent defect of memory B cell responses in treated patients. The decreased CCR6 expression that we observed in HIV-specific cTfh from treated patients also points to cTfh dysfunction, as low CCR6 expression may reflect perturbed differentiation (13) and may directly impair cTfh relocalization to infected tissues. The higher FAS receptor expression in specific cTfh from treated patients may also contribute to immune dysfunction, by priming cTfh-interacting cells for apoptosis. Taken together, the phenotypic analysis of HIV-specific cTfh highlighted signs of generalized immune activation in treated patients and of a more contained but nevertheless detectable activation in HIV controllers. The finding that HIV-specific cTfh are not quiescent in the latter group suggests that spontaneous HIV control relies on chronic stimulation of T follicular helper function, which may be needed to sustain high-level memory B cell responses.

Several studies have reported associations between the frequency of particular cTfh subsets and the breadth of circulating NAbs directed at HIV (23, 24, 72). These findings point to the need for a generally preserved cTfh population for the development of NAb breadth, which relies on continuous cycles of B cell maturation. A limitation of our study is that we did not include a control group of patients who acquired NAb breadth and who are thought to harbor a highly functional cTfh population. However, it is now apparent from several studies, including ours, that HIV controllers can develop potent memory B cell responses without high NAb titers (40, 55, 56) and that assessing the “silent” memory B cell compartment is needed to evaluate the status of the antiviral humoral response. The frequency of HIV-specific memory B cells still correlates with bNAb titers in a subset of HIV controllers who express the HLA-B57 allele (57), suggesting that in this particular group HIV-specific B cells have not returned to a quiescent memory state. Another study reported that the frequency of HIV-specific memory B cells decreased in HIV controllers who were given antiretroviral therapy, indicating that residual viral replication could still be a driver of memory B cell responses in the controller group (56). Our results suggest that another key determinant of the magnitude of memory B cell responses is the availability of T follicular help, as indicated by the positive correlation between HIV-specific cTfh frequency and HIV-specific IgG secretion by stimulated memory B cells. These findings are in line with a recent report where Tfh function defined functionally by IL-21 secretion in response to HIV peptides showed an association with Env-specific memory B cells (73). Genetic analyses of Env-specific IgG derived from controller memory B cells have been reported in a few cases and have revealed an unexpectedly high level of somatic hypermutation, reinforcing the notion of a continuous involvement of T follicular help in maintaining the controller humoral response (74).

Intriguingly, measurement of total IgG production suggested a perturbation of cTfh-B cell interactions in both groups of HIV-infected patients compared to healthy blood donors. The impairment was not generalized, but a subset of patients showed decreased IgG induction and plasmablast differentiation in cTfh/BL cocultures. The fact that these parameters could be restored by IL-6 supplementation suggested that cytokine imbalance could be involved. A possible explanation may lie in a shift in the Th1/Tfh balance, considering that these two CD4^+^ T cell populations depend on antagonist differentiation factors and that chronic viral infections are thought to lead to a pronounced Th1 bias (9). The demonstration that IL-2-dependent signaling inhibits cTfh function in HIV-infected patients is compatible with the notion that Th1-derived cytokines antagonize Tfh function (75). We previously documented the persistence of HIV-specific CD4^+^ T cells with advanced Th1 differentiation in HIV controllers, which could contribute to a general alteration of cTfh differentiation (76). The persistence of Th1 effectors was less prominent in the case of treated patients treated in the long term, but other parameters may perturb Tfh differentiation, such as abnormal immune activation or residual induction of type I interferon (77). We tested the effect of the IL-6 cytokine on cTfh function as several studies suggest an important contribution of this cytokine to Tfh-BL interactions. In particular, IL-6 produced by human plasmablasts was shown to promote the differentiation of Tfh *in vitro* and to stimulate the secretion of IL-21 by these cells (65). As IL-21 is thought to be a major mediator of Tfh help to B cells, this suggests a positive regulatory loop that promotes the differentiation of both cell types. The role of IL-6 on Tfh differentiation is supported by the decrease in cTfh observed in rheumatoid arthritis patients treated with an anti-IL-6R blocking antibody, by the role of IL-6 in driving Tfh amplification in murine models of chronic infection, and by the loss of the Tfh population in murine models deficient in B cells (9, 65, 78). Our finding that IL-6 restores total IgG production in patient cTfh/BL cocultures suggests that the IL-6/IL-21 regulatory loop functions suboptimally in both controllers and treated patients but that the defect is reversible rather than intrinsic.

In contrast, the analysis of Env-specific IgG production in the cTfh/BL coculture system revealed a defect in the treated patient group that could not be reversed by IL-6. Rather, the difference between groups persisted and was even amplified after IL-6 supplementation, with a >100-fold-lower production of Env-specific IgG in treated patient cocultures than in those of controllers. This loss of response may be explained by the irreversible exhaustion or loss of memory B cells, a notion supported by the low numbers of memory B cells able to bind Env-derived probes in treated patients (56). It was intriguing that Gag-specific IgG production did not show the same pattern but rather showed a defect that could be partly restored by IL-6 supplementation, pointing to the persistence of responsive memory B cells. Why Env- and Gag-specific memory B cell responses differ in severity of impairment remains to be elucidated, though one may speculate that Env, being a surface protein, is likely to be more frequently detected by B cell surface Ig receptors than the intracellular Gag protein, resulting in a more rapid exhaustion of Env-specific B cells. Also, it may be relevant that Env has the capacity to cross-link its receptor CD4, which was shown to impair TCR-dependent signaling and lead to CD4^+^ T cell anergy or apoptosis (79). This may perturb the development of T follicular help and indirectly lead to B cell exhaustion. Indeed, vaccination with a gp140 Env engineered to occlude the CD4 binding site resulted in earlier T cell responses and superior antibody and antibody-dependent cellular cytotoxicity (ADCC) responses compared to wild-type Env in a simian model (80). The inhibitory effect of wild-type Env may contribute to the generally lower CD4 responses to Env than to Gag and to the better prognostic value of Gag-specific T cell responses (60, 81). Whether Gag-specific Tfh help has a beneficial effect through the maturation of the Gag-specific antibody response is not known, as Gag-specific antibodies have not been shown so far to contribute significantly to HIV neutralization or to the elimination of HIV-infected cells. However, it is interesting that CD4 responses to Gag were more frequent in HIV-infected patients with detectable NAbs, while CD4 responses to Env gp120 were not predictive of neutralization (82). The fact that Gag-specific responses predicted the induction of Env-specific Nabs raised the possibility of intrastructural help, based on the idea that an Env-specific B cell internalizing a whole HIV virion after Env binding may process and present both Gag and Env peptides on MHC-II and may thus receive help by a Gag-specific CD4^+^ T cell. Intrastructural help has received experimental validation in mouse models, as shown in vaccination experiments where priming with Gag-specific DNA improved Env-specific antibody responses to a virus-like particle (VLP) HIV vaccine that contained both antigens (83). The adoptive transfer of Gag-primed CD4^+^ T cells was sufficient to improve the quality of VLP-induced Env-specific antibodies, emphasizing the key role of CD4 help in the development of antiviral humoral responses (84). Thus, CD4-dependent intrastructural help may account for the positive correlation that we observed between Gag-specific cTfh and the induction of Env-specific IgG.

In conclusion, controlled HIV infection was characterized by a high frequency of cTfh that showed signs of antigenic stimulation, pointing to an ongoing maturation of the humoral response. Functional assays revealed preserved cTfh-B cell interactions that sustained high-level HIV-specific memory B cell responses. These findings suggest that humoral immunity plays an underappreciated role in HIV control and that immunotherapeutic approaches that aim at a functional HIV cure should elicit potent Tfh function.

## MATERIALS AND METHODS

### Study design.

HIV controllers (HIC group; *n* = 15) were recruited through the CO21 CODEX cohort implemented by the Agence Nationale de Recherche sur le SIDA et les Hépatites Virales (ANRS). HIV controllers were defined as HIV-1-infected patients who had been seropositive for >5 years, who had received no antiretroviral treatment, and for whom >90% of plasma viral load measurements were undetectable by standard assays. All HIV controllers included in the study had current viral loads of <50 copies/ml. The group of efficiently treated patients (ART group; *n* = 15) had received antiretroviral therapy for a minimum of 5 years and showed long-term HIV-1 suppression with viral loads of <50 copies/ml. Treated patients were recruited at the Raymond Poincaré and Bicêtre hospitals (France). Patients were included in the tetramer study if their genotype matched at least one of the following alleles: DRB1*0101, DRB1*0401, DRB1*0405, DRB1*0701, DRB1*1101, DRB1*1302, or DRB1*1502 (see Table S1 in the supplemental material). Healthy donors were anonymous volunteers who donated blood at the Etablissement Français du Sang. The study was promoted by ANRS and approved by the Comité de Protection des Personnes IDF-VII under number 11-33. All participants gave written informed consent prior to inclusion in the study.

### Antibodies.

The following antibodies were used for cell surface staining: CD3-eFluor 780-allophycocyanin (eF780-APC; clone UCHT1), TCR-allophycocyanin (APC; clone IP26), and IL-2–APC (clone MQ1-17H12) (all from eBioscience); CD4-BD Horizon R phycoerythrin-CF594 (PE-CF594; clone RPA-T4), CD27-PE-Cy7 (Pe-Cy7-clone M-T271), CXCR5-Alexa Fluor 488 (AF488; clone RF8B2), and CCR7-Alexa Fluor 647 (AF647; clone 3D12) (all from BD Biosciences); CD14-Viogreen (clone TÜK4) and CD20-Viogreen (clone LT29) (from Miltenyi Biotec); and CD38-Alexa Fluor 700 (AF700; clone HIT2), CD20-peridinin chlorophyll protein (PerCP)/Cy5.5 (clone 2H7), CD8-brilliant violet 785 (BV785; clone RPA-T8), CD45RA-brilliant violet 421 (BV421; clone HI100), CCR7-PE-Cy7 (clone G043H7), and CD3-APC (clone SK7) (all from BioLegend). The fixable viability dye eFluor 506 (eF506; eBioscience) was added to restrict the analysis to live cells.

### Cell culture.

Peripheral blood mononuclear cells (PBMC) were isolated from heparinized blood via density gradient centrifugation on Ficoll-Paque Plus (GE Healthcare Life Sciences) and were either cryopreserved for future cell sorting or directly used for tetramer staining and cell surface phenotyping.

### MHC-II tetramer labeling and cTfh phenotyping.

Patients were genotyped for the HLA-DRB1 gene at a 4-digit resolution using the INNO-LiPA HLA-DRB1 Plus kit (Fujirebio). APC-labeled MHC-II tetramers loaded with Gag293 peptide (FRDYVDRFYKTLRAEQASQE) for the DRB1*0101, DRB1*0401, DRB1*0405, DRB1*0701, DRB1*1502, and DRB5*0101 alleles were obtained through the NIH Tetramer Core Facility at Emory University, Atlanta, GA. DRB1*1101 and DRB1*1302 biotinylated monomers were obtained through the Tetramer Core Laboratory of the Benaroya Research Institute (Seattle, WA). Monomers were loaded with 0.2 mg/ml peptide by incubation at 37°C for 72 h in the presence of 2.5 mg/ml *n*-octyl-β-d-glucopyranoside and protease inhibitors. Peptide-loaded monomers were tetramerized using APC-conjugated streptavidin (eBioscience). For each tetramer loaded with the Gag293 peptide, a corresponding control tetramer was loaded with an irrelevant peptide (the CLIP peptide PVSKMRMATPLLMQA).

For the *ex vivo* detection of Gag293-specific CD4^+^ T cells, patient PBMC were labeled with MHC-II tetramers as follows: 10^7^ PBMC were incubated with 1 µg MHC-II tetramer/10^6^ cells at a concentration of ≥1 µg/ml in complete RPMI medium supplemented with 15% human AB serum for 90 min at 4°C. Antibodies for surface markers were added for the last 30 min of labeling, using the following combination: CD3-eF780-APC, CD20-Viogreen, CD14-Viogreen, CD8-BV785, CD4 PE-CF594, CD45RA-BV421, CCR7-PE-Cy7, CXCR5-AF488, PD-1-PE, and Viability-eF506. Cells were washed twice with PBA (phosphate-buffered saline [PBS], 1% bovine serum albumin [BSA], 0.09% azide) and fixed using 2% paraformaldehyde (PFA) in PBS. Fluorescence was collected on an LSR Fortessa flow cytometer (BD Biosciences). All flow cytometry experiments were analyzed with FlowJo v8.8 software (Tree Star Inc.). Analysis was performed on singlet viable single cells, gated according to morphological criteria and viability dye expression.

For cTfh phenotyping, 1 × 10^6^ cells were stained in a total volume of 100 µl PBA with the same antibody panel as described above for 30 min at 4°C. Cells were washed twice with PBA, fixed in 2% PFA, and analyzed on an LSR Fortessa flow cytometer as described above.

### Antibody neutralization assay.

For neutralization, pseudoviruses were produced by cotransfecting 293-T cells with an HIV-1 Env expression plasmid (SF162.LS or MW965.26) and an Env-deficient HIV-1 backbone plasmid (pSG3ΔEnv). Infectious molecular clones of transmitted/founder viruses CH058, CH077, CH106, RHPA, THRO4156.18, REJO 4541.67, and TRJO4551.58 were obtained through the NIH AIDS Reagent Program. The corresponding viruses were produced by direct transfection of 293-T cells as previously described (57).

Plasma samples were tested for their ability to neutralize HIV-1 using the TZM-bl neutralization assay as described previously (85). Negative controls consisted of HIV-negative plasma from healthy blood donors. The presence of nonspecific neutralizing activity in patient plasma was tested by their capacity to neutralize the murine retrovirus MLV. Two tier 1 reference strains (SF162.LS, subtype B, and MW965.26, subtype C), six tier 2 transmitted/founder subtype B HIV-1 strains (CH058, CH077, CH106, RHPA, THRO4156.18, and REJO4541.67), and one tier 3 transmitted/founder subtype B HIV-1 strain (TRJO4551.58) were tested. The 50% inhibitory reciprocal dilution (IRD_50_) was defined as the reciprocal of the sample dilution that caused a 50% reduction in relative luminescence units (RLU).

### Fluorescence-activated cell sorting (FACS).

Following an overnight culture at a concentration of 5 × 10^6^ cells/ml, PBMC were washed with PBS-1% fetal bovine serum (FBS). Cells were then stained for 30 min at 4°C at 40 × 10^6^ cells/ml. The cell surface antibody panel consisted of the following antibodies: CD3-eF780-APC, CD20-Viogreen, CD8-BV785, CD4 PE-CF594, CD45RA-BV421, CCR7-AF647, CXCR5-AF488, and CD27-PE-Cy7. Stained samples were sorted using a FACSAria II cell sorter (BD Biosciences) installed in a microbiological safety cabinet. The collected cells were directly used for coculture assays.

### cTfh/BL coculture assays.

The cTfh cell population defined as CD3^+^ CD20^−^ CD4^+^ CD45RA^−^ CXCR5^+^, MemX5^−^ cells defined as CD3^+^ CD20^−^ CD4^+^ CD45RA^−^ CXCR5^−^, or naive CD4^+^ T cells (CD3^+^ CD20^−^ CD4^+^ CD45RA^+^ CCR7^+^) were sorted and plated in a 96-well plate in the presence of memory B cells (CD3^+^ CD20^+^ CD27^+^) at a 1:1 ratio (20 × 10^3^ cells each) in the presence of 1 µg/ml staphylococcal enterotoxin A (SEA) and 1 µg/ml staphylococcal enterotoxin E (SEE) in complete RPMI 1640 medium, in the presence or absence of IL-6 at 10 ng/ml (Miltenyi Biotec). B cells were analyzed for their phenotype at day 7 of coculture using the following antibody combination: CD3-APC, CD4 PE-CF594, CD20-PerCP-Cy5-5, CD38-AF700, CD27-PE-Cy7, and Viability-eF506. Fluorescence was collected on an LSR Fortessa flow cytometer (BD Biosciences). ELISAs were performed on supernatants collected at day 12 of coculture for the detection of total, p24-specific, and gp140-specific immunoglobulins.

### ELISAs.

Analysis of total IgG production in coculture supernatants was performed using the human IgG kit (GenWay, San Diego, CA) according to the manufacturer’s instructions. To detect Env-specific IgG production, we used as antigen the trimeric glycoprotein gp140 MN-LAI, which consists of the gp120 subunit from HIV-1 clone MN fused to the extracellular domain of HIV-1 LAI gp41 (66). Ninety-six-well Immulon 2HB plates (Thermo Scientific) were coated overnight at 4°C with 2.5 µg/ml of gp140 MN-LAI in PBS. The following day, plates were washed four times with PBS plus 0.05% Tween 20 plus 10 mM EDTA (PBST) and blocked for 2 h with PBST plus 5% FBS at room temperature (RT). Plates were subsequently washed, and the culture supernatants were added at decreasing dilutions for 2 h at RT. The plates were then washed and incubated with secondary anti-human IgG–horseradish peroxidase (HRP) at a concentration of 0.4 µg/ml (Jackson ImmunoResearch) for 1 h at RT. Plates were then thoroughly washed, and 100 µl of tetramethylbenzidine substrate (TMB; Sigma-Aldrich) was added until the appearance of color. The enzymatic reaction was stopped by the addition of 100 µl of 0.5 M H_2_SO_4_, and the optical density (OD) was then measured at a 450-nm wavelength. Env-specific IgG production was evaluated in equivalent (Eq) micrograms per milliliter, after normalization to a standard curve obtained with the human monoclonal antibody (MAb) 2G12.

To detect Gag-specific IgG, a recombinant p24 Gag protein produced in baculovirus (reagent number 12028 from the NIH AIDS Reagent Program) was used as a capture antigen. The p24 Gag protein was coated at 2 µg/ml in bicarbonate buffer (pH 9.6) on MaxiSorp 96-well plates (Nunc) overnight at 4°C. The ELISA was then carried out as described above. Gag-specific IgG production was evaluated in equivalent (Eq) micrograms per milliliter, after normalization to a standard curve obtained with an HIV controller plasma.

HIV-specific IgGs were detected in patient plasma as previously described (57). Briefly, 96-well plates were coated overnight in carbonate buffer with the following proteins: 1 µg/ml of sheep anti-human IgG (Binding Site, France) for detecting total IgGs, 1 µg/ml of gp140 or gp160 MN-LAI (86) for detecting Env-specific IgGs, 1 µg/ml of gp41 S30 (87) for detecting anti-anti-gp41 IgGs, and 0.5 µg/ml of Gag p24 protein (88) for detecting anti-p24 IgGs. After saturation with PBS containing 5% BSA, plates were incubated with diluted sera for 2 h at 37°C. IgGs were detected by addition of a secondary goat anti-human IgG conjugated to HRP, followed by incubation with the TMB substrate. Concentrations of IgGs were estimated according to an internal IgG standard.

### Statistical analyses.

Comparisons between groups (HD, HIC, and ART) were performed using a nonparametric two-tailed Mann-Whitney U test. For paired analyses, we used the nonparametric Wilcoxon matched-pair signed-rank test or the two-tailed paired *t* test. Correlations were analyzed with Spearman’s rank correlation coefficient. All statistical analyses were made using the Prism v6.0 software (GraphPad). Differences of *P* < 0.05 were considered significant. Horizontal bars represent medians in all cases.
