# Supplementary material for: A High Frequency of HIV-Specific Circulating Follicular Helper T Cells Is Associated with Preserved Memory B Cell Responses in HIV Controllers
Source: mBio. 2018 May 8;9(3):e00317-18. doi: 10.1128/mBio.00317-18 (PMC5941072; doi:10.1128/mBio.00317-18)
Supplement: FIG S1 [file mbo003183876sf1.pdf]

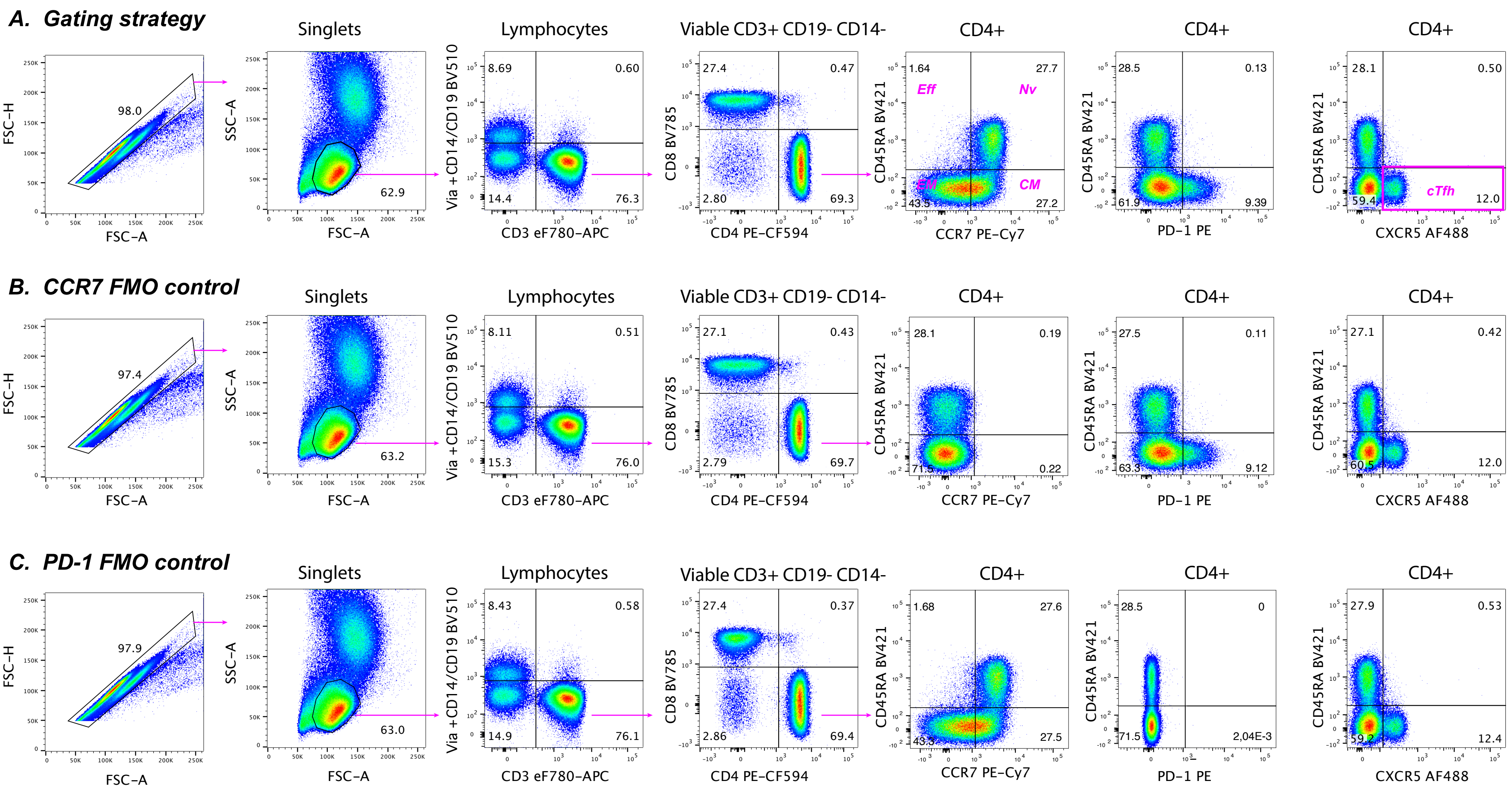

Supplemental Figure S1: Gating strategy and FMO controls for cTfh phenotyping

(A) CD4+ T cells were analyzed by gating the singlet viable CD14- CD19- CD3+ CD4+ lymphocyte population. The gate for the CCR7 marker was set on a CD45RA/CCR7 dot plot, which enabled a clear distinction between naive (Nv; CD45RA+ CCR7+) and memory subsets. The three memory subsets, including the central memory (CM; CDRA- CCR7+), effector memory (EM; CD45RA- CCR7-), and advanced effector (Eff; CD45RA+ CCR7-) subsets are reported. Similarly, gates for the PD-1 and CXCR5 markers were set on dot plots in function of the CD45RA parameter, which enabled a clear distinction between the positive and negative populations (2 rightmost dot plots).

(B, C) "Fluorescence minus one" (FMO) controls were obtained by omitting a single antibody from the labeling antibody cocktail. Analysis of FMO controls for CCR7 (B) and PD-1 (C) showed that omitting one marker did not significantly change the frequency of positive cells for other markers, confirming the validity of the compensation matrix and of the gating strategy.
