# Supplementary material for: A High Frequency of HIV-Specific Circulating Follicular Helper T Cells Is Associated with Preserved Memory B Cell Responses in HIV Controllers
Source: mBio. 2018 May 8;9(3):e00317-18. doi: 10.1128/mBio.00317-18 (PMC5941072; doi:10.1128/mBio.00317-18)
Supplement: TABLE S1 [file mbo003183876st1.pdf]

A

Patient group	Duration of infection years	Viral load HIV-1 copies/mL	CD4 /mm3	Nadir of CD4+ T cells /mm3	Duration of antiretroviral treatment
HIC	16.3 [7.6 - 27.7]	< 50	944 [315 - 1333]	N/A	N/A
ART	13.8 [8.9 - 27.8]	< 50	615 [211 - 987]	64 [10 - 450]	9.5 [6.4 - 26.1]
P value* HIC vs ART	N.S.	N.S.	P<0.01	-	-

B

Patient ID	Duration of infection years	Viral load HIV-1 copies/mL	CD4 /mm3	HLA-DR typing	HLA-DR tetramer used
**HIC01**	23.9	<50	1333	DR1, DR11	DR11
**HIC02**	16.3	<50	944	DR11, DR12	DR11
**HIC03**	9.0	<50	1252	DR14, DR16, DRB5	DRB5
**HIC04**	27.4	<50	1030	DR15, DRB5	DRB5
**HIC05**	27.1	<50	1000	DR11, DR15, DRB5	DR11
**HIC06**	10.8	<50	1016	DR8, DR13	DR13
**HIC07**	8.3	<50	755	DR13, DR15, DRB5	DRB5
**HIC08**	11.6	<50	744	DR7, DR15, DRB5	DRB5
**HIC09**	20.0	<50	315	DR1, DR7	DR7
**HIC10**	14.9	<50	1063	DR1, DR7	DR1
**HIC11**	11.7	<50	747	DR9, DR13	
**HIC12**	27.7	<50	929	DR14, DR15, DRB5	
**HIC13**	21.7	<50	765	DR3, DR8	
**HIC14**	18.9	<50	702	DR1, DR16	
**HIC15**	7.6	<50	1190	DR3/DR7	
Median	16.3	<50	944.0		

C

Patient ID	Duration of infection years	Viral load HIV-1 copies/mL	CD4 /mm3	HLA-DR typing	HLA-DR tetramer used
**ART01**	9.0	<50	427	DR1, DR3	DR1
**ART02**	19.0	<50	411	DR3, DR4	DR4
**ART03**	8.9	<50	424	DR1, DR15, DRB5	DR1
**ART04**	11.9	<50	417	DR15, DR16, DRB5	DRB5
**ART05**	27.3	<50	615	DR1, DR7	DR1
**ART06**	13.8	<50	211	DR15, DR16, DRB5	DR1
**ART07**	25.9	<50	403	DR4, DR7	DR4*
**ART08**	12.8	<50	754	DR9, DR16, DRB5	DRB5
**ART09**	18.9	<50	328	DR1, DR13	
**ART10**	15.4	<50	965	DR4, DR13	
**ART11**	12.3	<50	987	DR1, DR1	
**ART12**	27.8	<50	860	DR4, DR13	
**ART13**	9.2	<50	730	DR7, DR13	
**ART14**	9.7	<50	948	DR1, DR14	
**ART15**	26.9	<50	754	DR8, DR16	
Median	13.8	<50	615		

Supplemental Table S1: Clinical characteristics and HLA-DR typing of patients included in the study

15 HIV controllers (HIC) and 15 treated patient (ART) were included in the study.
(A) Summary of clinical characteristics of studied patients. Median values and ranges are reported. * P values estimated with the Mann-Whitney U test are reported. N.S.: not significant. N/A: not available.
(B, C) Clinical characteristics and HLA-DR typing of HIC (B) and ART (C) patients.
Patients included in the study were genotyped for HLA-DRB1. A subgroup of 10 HIC and 8 ART were analyzed with HLA-DR-matched MHC II tetramers loaded with the Gag293 peptide. The tetramers used for this analysis is reported in the rightmost column. The following tetramers were used: HLA DRB1*0101 (DR1),DRB1*00401 (DR4), DRB1*0405 (DR4*), DBRB1*0701 (DR7), DRB1*1101 (DR11), DRB1*1302 (DR13), DRB1*1502 (DR15), and DRB5*0101 (DRB5).
