# Supplementary material for: A High Frequency of HIV-Specific Circulating Follicular Helper T Cells Is Associated with Preserved Memory B Cell Responses in HIV Controllers
Source: mBio. 2018 May 8;9(3):e00317-18. doi: 10.1128/mBio.00317-18 (PMC5941072; doi:10.1128/mBio.00317-18)
Supplement: FIG S2 [file mbo003183876sf2.pdf]

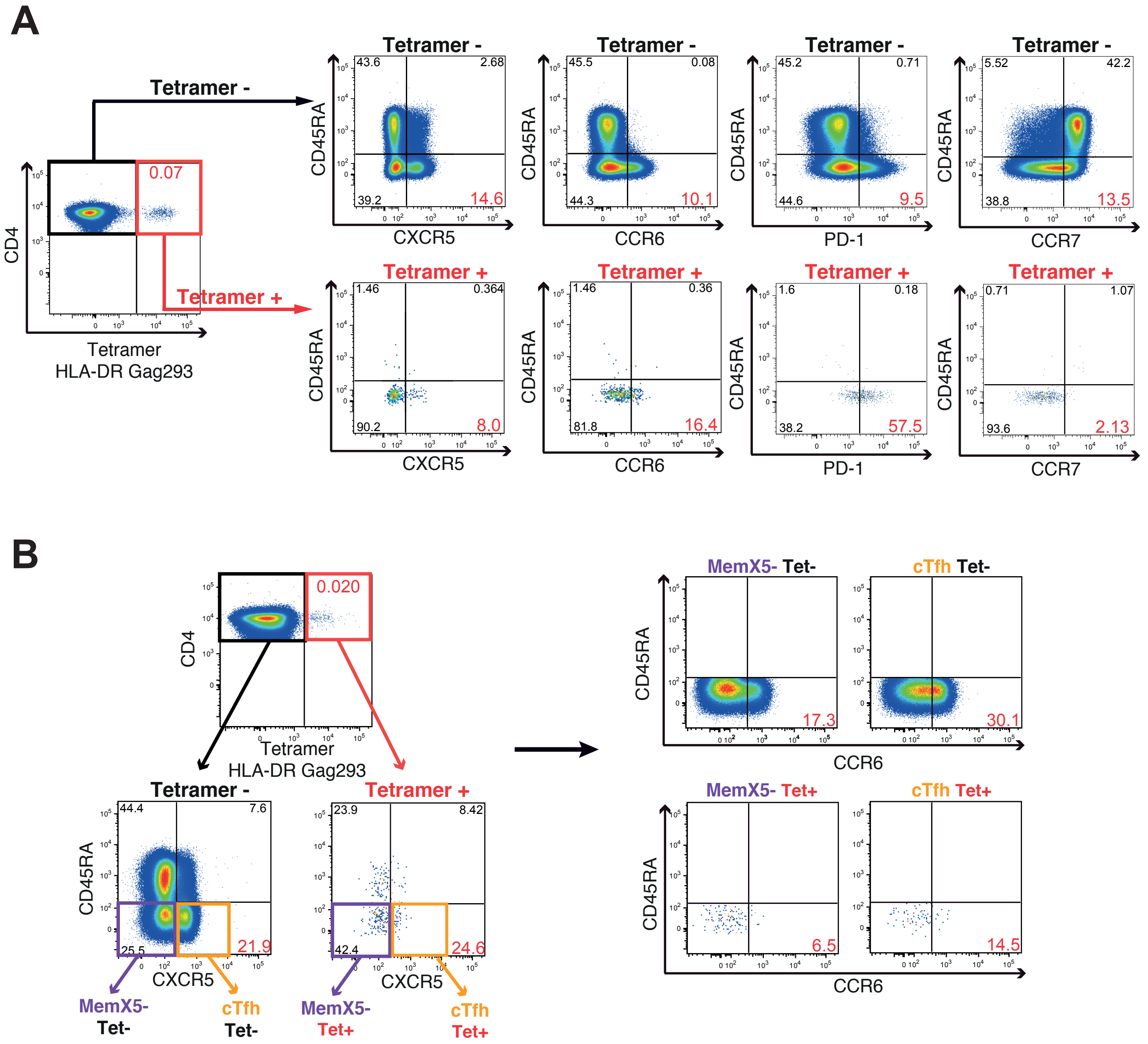

Supplemental Figure S2: Gating strategy for the analysis of HIV-specific CD4+ T cell populations

(A) Example of gating used to analyze HIV-specific CD4+ T populations in one HIV controller.
Left: HIV-specific CD4+ T cells were detected by labeling with an MHC-II tetramer loaded with the Gag293 peptide.
Right: Plots depicting how the gates were set for the analysis of CCR6, PD-1, and CCR7 expression in the Gag293-specific (bottom row) and non-specific (top row) CD4+ T cells populations. The CXCR5, CCR6, PD-1 and CCR7 gates were set based on differential expression in the CD45RA+ and CD45RA- populations

(B) Example of CCR6 expression analysis in Gag293-specific and non-specific CD4+ T cells from a treated patient.
Left: Gating strategy used to analyze HIV-specific CD4+ T cells. Top: plot illustrating the detection of Gag293-specific CD4+ T cells by MHC-II tetramer labeling in a treated patient. Bottom: Gates used to select the Mem X5- (CD45RA- CXCR5-; purple) and cTfh (CD45RA- CXCR5+; orange) subsets in Gag293-specific (Tetramer+) and non-specific (Tetramer-) CD4+ T cells populations.
Right: Plots comparing CCR6 expression in the MemX5- (right) and cTfh (left) subsets, in both the non-specific (Tet-, top) and specific (Tet+, bottom) CD4+ T cell populations.
