# Supplementary material for: A High Frequency of HIV-Specific Circulating Follicular Helper T Cells Is Associated with Preserved Memory B Cell Responses in HIV Controllers
Source: mBio. 2018 May 8;9(3):e00317-18. doi: 10.1128/mBio.00317-18 (PMC5941072; doi:10.1128/mBio.00317-18)
Supplement: FIG S3 [file mbo003183876sf3.pdf]

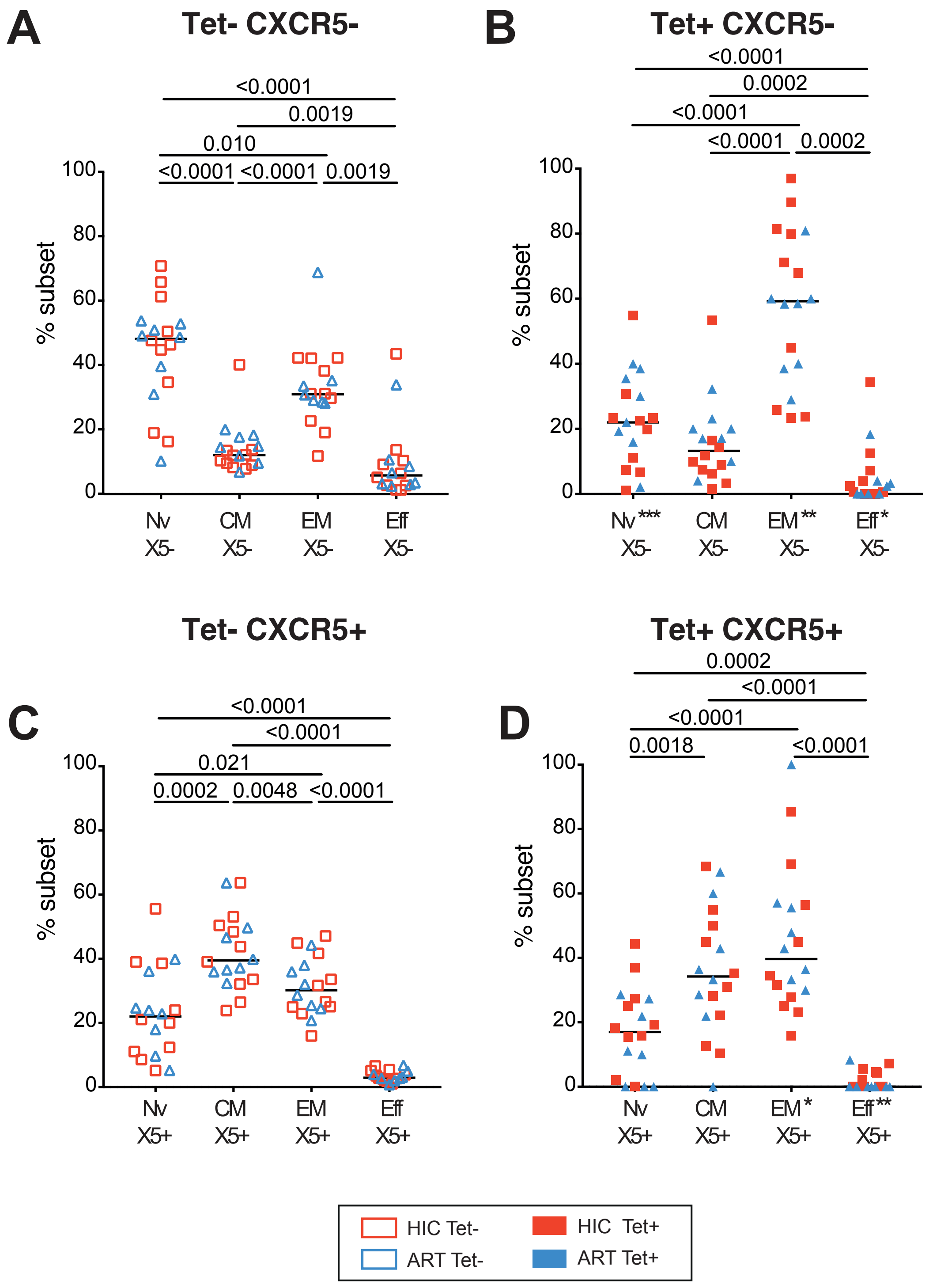

Supplemental Figure S3: Distribution of naive and memory subsets in Gag293-specific and non-specific CD4+ T cells differs depending on CXCR5 expression

Gag293-specific (Tet+) and non-specific (Tet-) CD4+ T cells expressing CXCR5 (X5+) or not (X5-) were analyzed for the distribution of the 4 following subsets: Naive (Nv; CD4RA+ CCR7+), central memory (CM; CD45RA- CCR7+), effector memory (EM; CD45RA- CCR7-), and effector (Eff; CD45RA+ CCR7-). Analyzes were carried out in cells from HIV Controllers (HIC, n=10) and treated patients (ART, n=8). As no significant differences were found in subset distribution between the HIC and ART groups, data from both groups were pooled and plotted together.
(A) Non-specific CXCR5- CD4+ T cells. (B) Gag293-specific CXCR5- CD4+ T cells.
(C) Non-specific CXCR5+ CD4+ T cells. (D) Gag293-specific CXCR5+ CD4+ T cells.
P-values for significant differences (P<0.05) obtained by the Mann-Whitney U test between subsets on the same graph are reported on each graph. Significant inter-graph differences obtained by the Mann-Whitney U Test between Tet- and Tet+ matching subsets are indicated by stars next to the subset name on panel C (Tet- X5- vs Tet+ X5-) or D (Tet- X5+ vs Tet+ X5+): * p-<0.05; **p<0.01; ***p<0.001.
