# Supplementary material for: A High Frequency of HIV-Specific Circulating Follicular Helper T Cells Is Associated with Preserved Memory B Cell Responses in HIV Controllers
Source: mBio. 2018 May 8;9(3):e00317-18. doi: 10.1128/mBio.00317-18 (PMC5941072; doi:10.1128/mBio.00317-18)
Supplement: FIG S4 [file mbo003183876sf4.pdf]

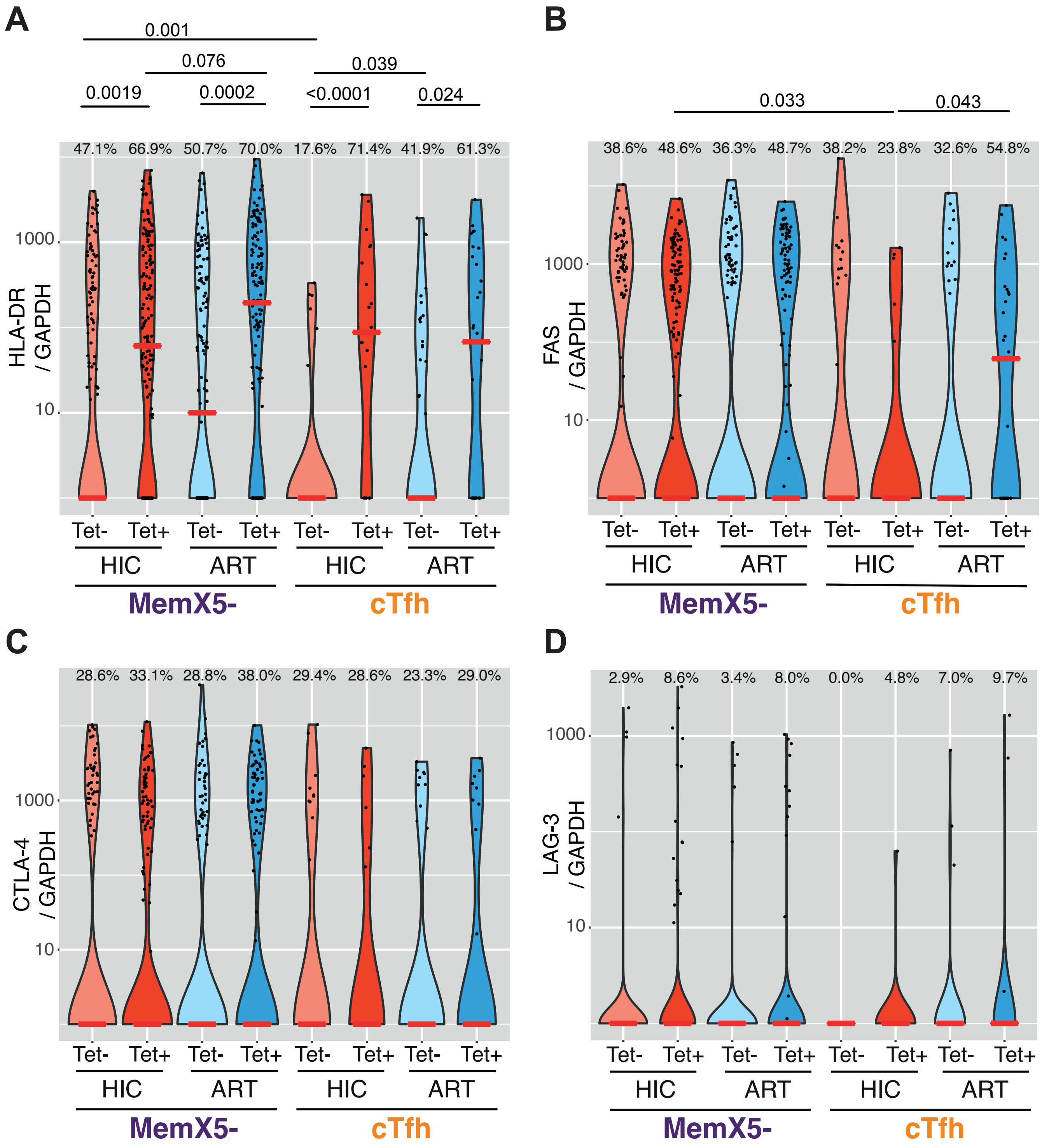

Supplemental Figure S4: Expression of activation and exhaustion markers in HIV-specific and non-specific CD4+ T cell subsets

(A) HLA-DR, (B) FAS, (C) CTLA-4, and (D) LAG-3 mRNA expression was measured in sorted Gag293-specific (Tet+) and non-specific (Tet-) CD4+ T cell subsets. Reverse-transcribed mRNA was quantitated at the single cell level by quantitative real-time PCR on a microfluidics C1 chip (Fluidigm), as per manufacturer's instructions. Gene expression normalized to that of the housekeeping gene GAPDH and multiplied by a factor 10,000 is reported. The analysis was carried out on cells collected from 9 HIC and 9 ART patients. The number of cells analyzed was ≥140 for each group in the MemX5 subset and ≥ 21 for each group in the cTfh subset. Violin plots visualize the distribution of the dataset. Median values are indicated by red bars. The percentage of positive cells (with a normalized gene expression >10) is indicated above each plot. P values obtained by the Mann-Whitney U test are reported.
