# Supplementary material for: A High Frequency of HIV-Specific Circulating Follicular Helper T Cells Is Associated with Preserved Memory B Cell Responses in HIV Controllers
Source: mBio. 2018 May 8;9(3):e00317-18. doi: 10.1128/mBio.00317-18 (PMC5941072; doi:10.1128/mBio.00317-18)
Supplement: FIG S5 [file mbo003183876sf5.pdf]

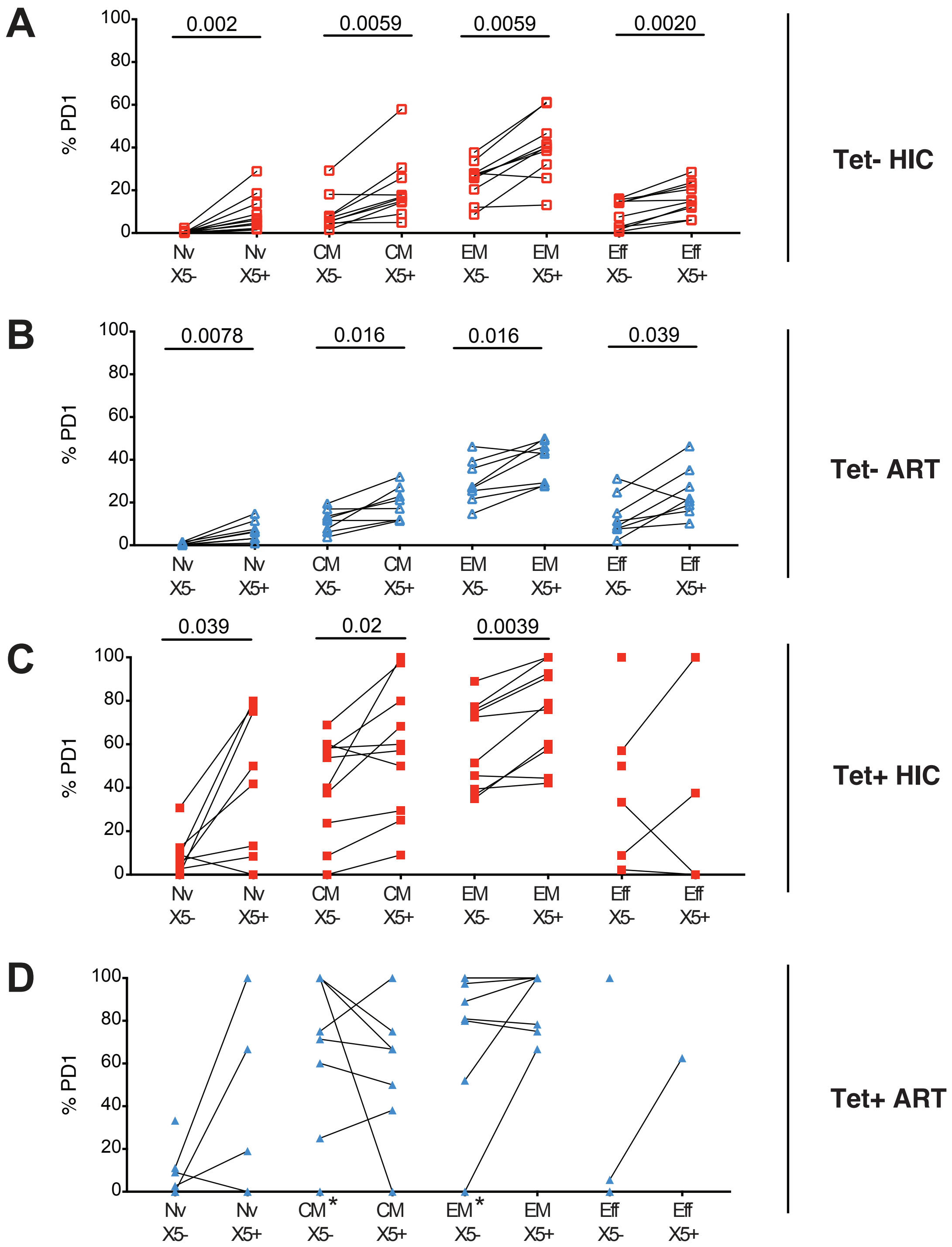

Supplemental Figure S5: PD-1 expression in naive and memory subsets of Gag293-specific and non-specific CD4+ T cells differs depending on CXCR5 expression

The frequency of PD-1 expressing cells was analyzed in subsets of Gag293-specific (Tet+) and non-specific (Tet-) CD4+ T cells expressing CXCR5 (X5+) or not (X5-). PD-1 expression was measured in the 4 following subsets: Naive (Nv; CD4RA+ CCR7+), central memory (CM; CD45RA- CCR7+), effector memory (EM; CD45RA- CCR7-), and effector (Eff; CD45RA+ CCR7-). Analyzes were carried out in cells from HIV controllers (HIC, n=10) and treated patients (ART, n=8). Tet+ Nv and Eff data points with too few cells for analysis are not represented.
(A) Non-specific CD4+ T cells from HIC patients. (B) Non-specific CD4+ T cells from ART patients.
(C) Gag293-specific CD4+ T cells from HIC patients. (D) Gag293-specific CD4+ T cells from ART patients.
P-values for significant differences (P<0.05) obtained by the Wilcoxon matched-pairs test between X5- and X5+ matched subsets are reported on each graph. Significant inter-graph differences obtained by the Mann-Whitney U Test between Tet+ HIC and Tet+ ART matching subsets are indicated by stars next to the subset name on panel D: * p<0.05.
