# Supplementary material for: A High Frequency of HIV-Specific Circulating Follicular Helper T Cells Is Associated with Preserved Memory B Cell Responses in HIV Controllers
Source: mBio. 2018 May 8;9(3):e00317-18. doi: 10.1128/mBio.00317-18 (PMC5941072; doi:10.1128/mBio.00317-18)
Supplement: FIG S6 [file mbo003183876sf6.pdf]

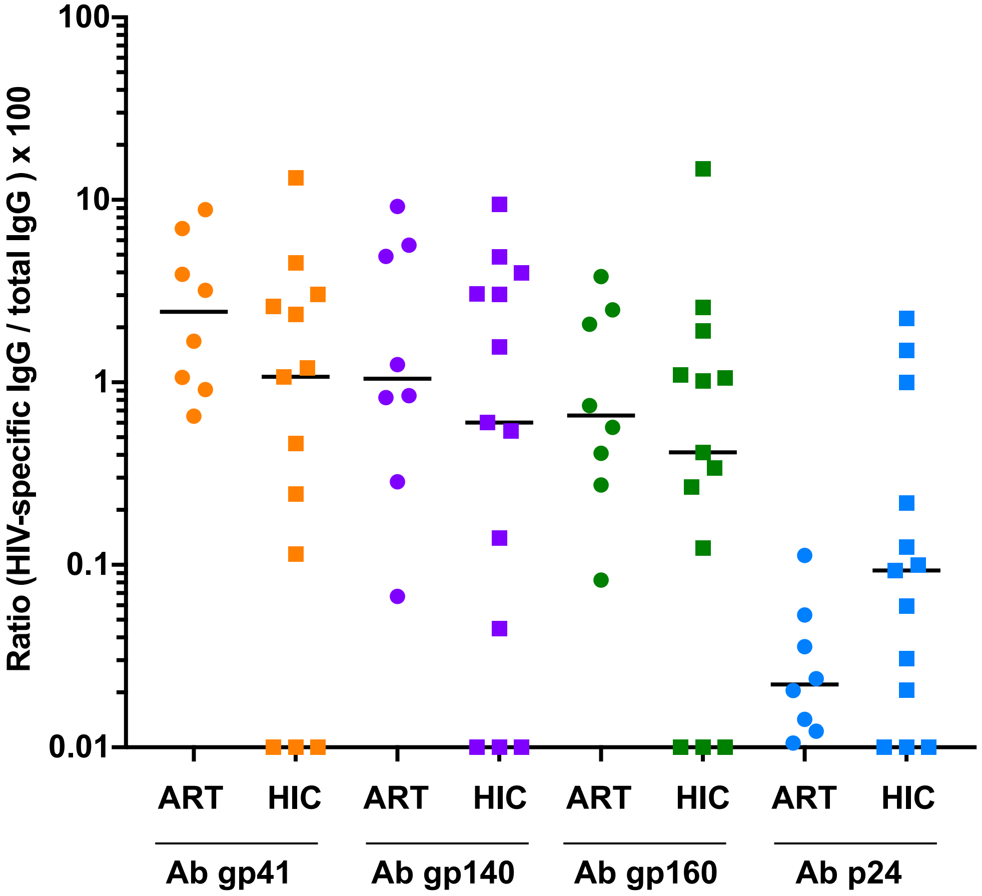

Supplemental Figure S6: HIV-specific antibodies in patient plasma

Antibodies specific for HIV-1 gp41 S30, gp140 and gp160 MN/LAI, and p24 Gag were measured by ELISA in patient plasma. The ratio of HIV-specific antibody concentration to the total IgG concentration in plasma, multiplied by 100 is reported. Ratios for undetectable HIV-specific antibodies were assigned a threshold value of 0.01. Median values are indicated by black bars.
