# Supplementary material for: A High Frequency of HIV-Specific Circulating Follicular Helper T Cells Is Associated with Preserved Memory B Cell Responses in HIV Controllers
Source: mBio. 2018 May 8;9(3):e00317-18. doi: 10.1128/mBio.00317-18 (PMC5941072; doi:10.1128/mBio.00317-18)
Supplement: TABLE S2 [file mbo003183876st2.pdf]

Supplemental Table S2 : Neutralizing capacity of HIV controller plasma

A- Plasma samples obtained at inclusion in the current study

		Tier 1		Tier 2						Tier 3	Control
**Patient ID**	**Month**	**SF162**	**MW965.26**	**CH058**	**CH077**	**CH106**	**RHPA**	**THRO**	**REJO**	**TRJO**	**MLV**
**HIC01**	**M1**	**6131**	**2283**	<10	<10	<10	<10	<10	<10	<10	<10
**HIC02**	**M1**	<10	<10	<10	<10	<10	<10	<10	<10	<10	<10
**HIC04**	**M1**	**7385**	**4342**	<10	<10	<10	<10	<10	<10	<10	<10
**HIC05**	**M1**	**9738**	**3492**	<10	<10	<10	<10	<10	<10	<10	<10
**HIC06**	**M1**	**1037**	**5164**	**69**	**96**	<10	**67**	<10	<10	<10	<10
**HIC07**	**M1**	**139**	**789**	<10	<10	<10	<10	<10	<10	<10	<10
**HIC08**	**M1**	**1019**	**902**	<10	31	<10	<10	<10	<10	<10	<10
**HIC10**	**M1**	<10	<10	<10	<10	<10	<10	<10	<10	<10	<10
**HIC11**	**M1**	**4911**	**3830**	<10	<10	<10	<10	<10	<10	<10	<10
**HIC14**	**M1**	**58**	**164**	<10	<10	<10	<10	<10	<10	<10	<10
**HIC15**	**M1**	<10	**94**	<10	<10	<10	<10	<10	<10	<10	<10

B- Duplicate plasma samples collected in a previous study

		Tier 1		Tier 2						Tier 3	Control
**Patient ID**	**Month**	**SF162**	**MW965.26**	**CH058**	**CH077**	**CH106**	**RHPA**	**THRO**	**REJO**	**TRJO**	**MLV**
**HIC02**	**M-44**	10	ND	<10	<10	<10	<10	<10	<10	<10	<10
**HIC04**	**M-30**	**1000**	ND	10	10	10	10	<10	<10	<10	10
**HIC06**	**M-8**	**500**	ND	**80**	**80**	<10	**110**	<10	<10	<10	10
**HIC08**	**M-4**	**300**	ND	<10	<10	<10	<10	<10	<10	<10	<10
**HIC11**	**M-28**	**500**	ND	10	10	<10	<10	<10	<10	<10	10

Titers of HIV neutralizing antibodies were determined in a TZM-bl-based assay. Patients plasma were tested for their capacity to neutralize 2 Tier 1 strains, 6 Tier 2 strains, 1 Tier 3 strain, and 1 control murine leukemia virus (MLV) MLV strain. The reciprocal of the inhibitory dilution 50 (ID50) is reported. Values >10 are considered significant.
(A) Neutralizing titers obtained at inclusion in the study.
(B) Neutralizing titers obtained for a subset of the same patients in retrospective plasma samples. The month of plasma collection is reported in the second column. ND: not done.
