# Supplementary material for: A High Frequency of HIV-Specific Circulating Follicular Helper T Cells Is Associated with Preserved Memory B Cell Responses in HIV Controllers
Source: mBio. 2018 May 8;9(3):e00317-18. doi: 10.1128/mBio.00317-18 (PMC5941072; doi:10.1128/mBio.00317-18)
Supplement: FIG S7 [file mbo003183876sf7.pdf]

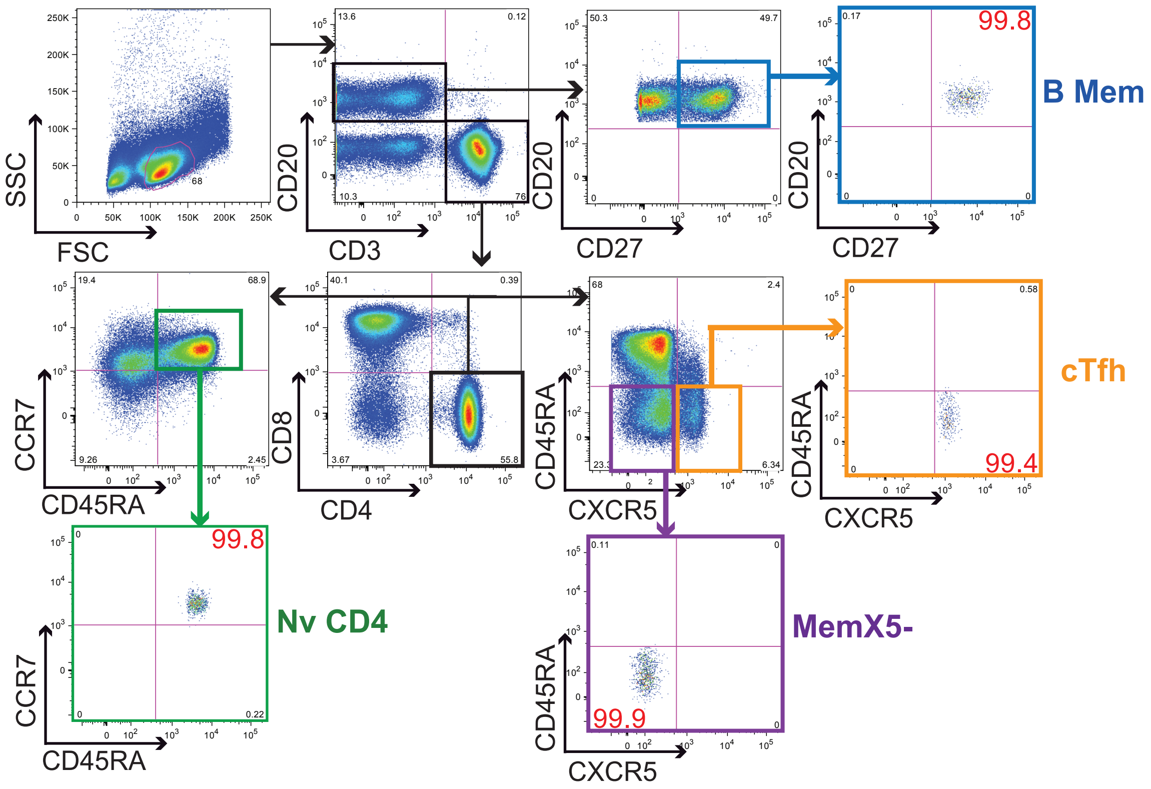

Supplemental figure S7: Gating strategy used for sorting CD4+ T cell and B cell subsets. Flow cytometry plots showing the gating strategy used for sorting cTfh cells (CD3+ CD20- CD4+ CD45RA- CXCR5+; orange), MemX5-(CD3 CD20- CD4+ CD45RA- CXCR5-; purple), naive CD4+ T cells (CD3+ CD20- CD4+ CD45RA+ CCR7+; green), and memory B cells (CD3- CD20+ CD27+; blue) among live singlet PBMC. A representative example from a healthy donor sample pre- and post-sort is shown. Post-sort sample plots are outlined with the color corresponding to the different cellular subsets.
