# Supplementary material for: A High Frequency of HIV-Specific Circulating Follicular Helper T Cells Is Associated with Preserved Memory B Cell Responses in HIV Controllers
Source: mBio. 2018 May 8;9(3):e00317-18. doi: 10.1128/mBio.00317-18 (PMC5941072; doi:10.1128/mBio.00317-18)
Supplement: FIG S8 [file mbo003183876sf8.pdf]

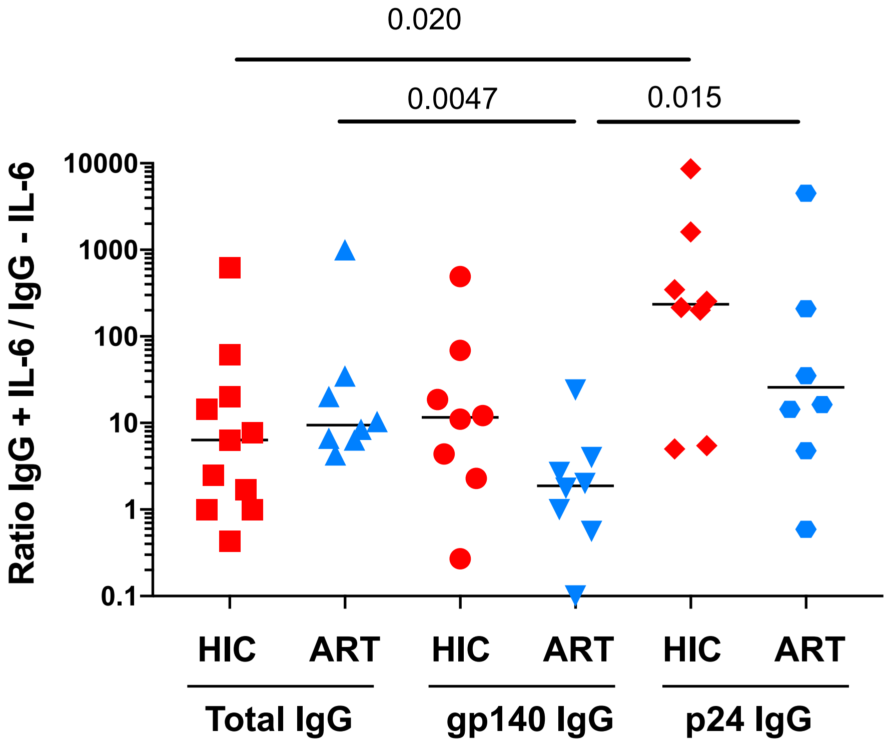

Supplemental Figure S8: Effect of IL-6 on IgG secretion in cTfh/B cell cocultures

cTfh cells and memory B cells were cocultivated in the presence of superantigens SEA + SEE for 12 days. Supernatants were assessed for the presence of total IgG, IgG specific for HIV Env gp140, and IgG specific for HIV capsid protein Gag p24. The ratio of IgG obtained with IL-6 supplementation to IgG obtained without IL-6 is reported for cocultures of cells from HIV controllers (HIC) and treated patients (ART). Significant differences (P<0.05) obtained withe the Mann-Whitney U test are reported.
